# Evidence of Adaptive Evolution in *Wolbachia*-Regulated Gene DNMT2 and Its Role in the Dipteran Immune Response and Pathogen Blocking

**DOI:** 10.3390/v13081464

**Published:** 2021-07-27

**Authors:** Tamanash Bhattacharya, Danny W. Rice, John M. Crawford, Richard W. Hardy, Irene L. G. Newton

**Affiliations:** 1 Department of Biology, Indiana University Bloomington, Bloomington, IN 47405, USA; tbhattac@fredhutch.org (T.B.); danny.w.rice@gmail.com (D.W.R.); johmcraw@indiana.edu (J.M.C.); 2 Basic Sciences Division, Fred Hutchinson Cancer Research Center, Seattle, WA 98109, USA

**Keywords:** methyltransferase, adaptive evolution, diptera, drosophilidae, culicidae, virus, wolbachia

## Abstract

Eukaryotic nucleic acid methyltransferase (MTase) proteins are essential mediators of epigenetic and epitranscriptomic regulation. DNMT2 belongs to a large, conserved family of DNA MTases found in many organisms, including holometabolous insects such as fruit flies and mosquitoes, where it is the lone MTase. Interestingly, despite its nomenclature, DNMT2 is not a DNA MTase, but instead targets and methylates RNA species. A growing body of literature suggests that DNMT2 mediates the host immune response against a wide range of pathogens, including RNA viruses. Curiously, although DNMT2 is antiviral in *Drosophila*, its expression promotes virus replication in mosquito species. We, therefore, sought to understand the divergent regulation, function, and evolution of these orthologs. We describe the role of the *Drosophila*-specific host protein IPOD in regulating the expression and function of fruit fly DNMT2. Heterologous expression of these orthologs suggests that DNMT2′s role as an antiviral is host-dependent, indicating a requirement for additional host-specific factors. Finally, we identify and describe potential evidence of positive selection at different times throughout DNMT2 evolution within dipteran insects. We identify specific codons within each ortholog that are under positive selection and find that they are restricted to four distinct protein domains, which likely influence substrate binding, target recognition, and adaptation of unique intermolecular interactions. Collectively, our findings highlight the evolution of DNMT2 in Dipteran insects and point to structural, regulatory, and functional differences between mosquito and fruit fly homologs.

## 1. Introduction

RNA virus inhibition by the arthropod endosymbiont *Wolbachia pipientis* is widely perceived as an effective biological vector control method. Moreover, recent reports regarding the successful deployment of this strategy in field trials around the globe are likely to continue to lead to a more widespread application [1]. As such, efforts to understand the molecular mechanism of *Wolbachia-*mediated pathogen blocking are underway, with recent findings highlighting the roles of cellular stress and metabolic pathways, in addition to those involved in RNA binding and processing [2,3,4,5]. In our previous study, we reported the role of the fruit fly gene DNA methyltransferase 2 (DNMT2), a gene known to function at the interface of these aforementioned cellular processes, as an essential determinant of endosymbiont-mediated inhibition of the prototype alphavirus, Sindbis (SINV, Togaviridae) [6]. Notably, at the time of this finding, the *Aedes* ortholog of DNMT2 was known to play a similar regulatory role within the context of *Wolbachia-*colonized mosquitoes infected with a different RNA virus, Dengue (DENV, Flaviviridae), thus suggesting the possibility that it may function as a mediator of host-pathogen interactions across multiple arthropod families [7].

Cellular DNA and RNA methyltransferases (MTases) are key mediators of epigenetic and epitranscriptomic regulation in eukaryotes. The former is carried out by a conserved family of DNA cytosine methyltransferases (DNMTs). The DNMT family includes true DNA MTases such as DNMT1, DNMT3A, DNMT3B, and DNMT3L [8,9]. The remaining member of the DNMT family is DNA MTase 2, or DNMT2, which, despite its name and sequence similarity to other DNMTs, has been demonstrated to have only minimal DNA methylation activity in vitro. Instead, it has been shown that DNMT2 binds and methylates RNA substrates in vivo and in vitro, thus classifying it as a novel class of RNA MTases [9,10,11]. Homologs of DNMT2 are present in the vast majority of animal, fungal, and plant species. Notably, DNMT2 is the only known DNMT present in dipteran insects such as *Drosophila melanogaster*, *Aedes aegypti*, *Aedes albopictus*, *Culex quinquefasciatus*, and *Anopheles gambiae* [12]. By extension, it is conceivable that all members of Drosophila and Culicidae families are DNMT2-only organisms.

Consistent with DNMT2′s role as a bona fide RNA MTase, evidence of genome-wide CpG methylation is nearly absent in these dipteran species, leaving the biological role of this MTase unclear [12,13]. Past studies investigating the biological function of DNMT2 suggest that it functions as a predominantly cytoplasmic protein during cellular stress and can lead to increased survival under stress conditions [14,15]. Under these conditions, DNMT2 is responsible for methylating transfer RNAs (e.g., tRNA_ASP_, tRNA_GLU_), a modification that aids in protecting these RNA species from stress-induced degradation [14,15,16]. Aside from these known functions, the role of DNMT2 in the immune response is a relatively recent finding, following reports of its role in regulating the silencing of retrotransposons that otherwise contribute to cell stress [17,18,19,20]. Furthermore, proper functioning of DNMT2 in *Drosophila melanogaster* is required for efficient Dicer-2 activity and, thus, by extension, the RNA interference pathway [21]. On its own, fruit fly DNMT2 inhibits several RNA viruses and protects the host against pathogenic bacteria [6,17,22]. Furthermore, DNMT2 orthologs of several other arthropods are involved in the colonization by pathogenic bacteria (*Helicoverpa armigera*), RNA viruses (*Aedes aegypti*, *Aedes albopictus*), and Plasmodium (*Anopheles albimanus*). Indeed, previous studies have demonstrated the roles of both *Drosophila melanogaster* and *Aedes* DNMT2 orthologs in regulating RNA virus infection. Notably, while DNMT2 in the fruit fly is responsible for limiting virus replication and production of infectious virus progeny, the *Aedes* orthologs seemingly play a proviral role in the mosquito host [7].

Given that they are known to differentially influence virus infection, we compared DNMT2 orthologs from *Drosophila melanogaster* (*Dm*DNMT2) and *Aedes albopictus* (*Aa*DNMT2) to identify differences in ortholog structure and regulation [6,7]. We found distinct differences in primary and tertiary protein structures between *Dm*DNMT2 and *Aa*DNMT2 that extend to other members of their respective families. Additionally, our findings suggest a distinct model of regulation for *Dm*DNMT2 expression in fruit flies. Unlike Aedes DNMT2, whose expression is thought to be under the control of host miRNAs, we present evidence suggesting that regulation of *Dm*DNMT2 expression and antiviral function depend on the fly protein interaction partner of DNMT2 or IPOD, which is unique to Drosophila, and seemingly absent within Culicidae. In light of these findings, we tested whether the antiviral function of *Dm*DNMT2 is due to intrinsic features of this MTase ortholog or whether it is dependent on the host. To this end, we performed heterologous expression of both Drosophila and Aedes DNMT2 in mosquito and fly cells, respectively, and assessed whether their ability to function as pro- or antiviral was dependent on the host context. Expression of both DNMT2 orthologs led to significant virus inhibition in fly cells, suggesting a host-driven inhibitory effect of increased MTase expression/activity. In contrast, expression of *Dm*DNMT2 in mosquito cells had neither an anti- nor proviral effect on virus proliferation, suggesting missing intermolecular interactions required for proper antiviral function in the non-native host background.

Our findings, and that of others, suggest that both fruit fly and mosquito DNMT2 function in host-pathogen interactions [6,7,17,22,23]. Genes involved in host immunity may face intense selective pressure, which manifests in the form of rapid evolution, often reflected by signatures of positive selection in the genome (e.g., Relish (Imd pathway) [24], although see [25] for a constraint in immune gene evolution). This selection then leads to the rapid, adaptive evolution of these genes and their encoded products, driven by intermolecular interactions between the protein and its target, e.g., pathogen-associated molecular patterns (PAMPs). Given its recently identified role in arthropod immunity, we hypothesized that recurrent host-pathogen interactions had impacted the molecular evolution of DNMT2 in Dipteran insects. In light of their well-documented history of harboring pathogens such as RNA viruses, we focused our analyses on members of Culicidae and Drosophila [6,7,17,23]. Consistent with our hypotheses, we found significant evidence of adaptive evolution along the ancestral lineages to all Dipteran DNMT2s as well as among DNMT2 orthologs of several members of the two aforementioned Dipteran families. Several amino acid positions within Drosophilid and Culicidae DNMT2 show evidence of rapid evolution. These residues were present within functionally important motifs, thus likely altering substrate binding and catalytic function. Additionally, the vast majority of sites were surface exposed, indicating that they may be involved in intermolecular interactions with cognate partners present within Drosophila or Culicidae. Collectively, our results provide evidence of divergent function and evolution of DNMT2 in dipterans, underscoring the importance of this otherwise non-canonical immune gene in host-pathogen interactions.

## 2. Materials and Methods

### 2.1. Insect and Mammalian Cell Culture

*Drosophila melanogaster* cells (JW18) with and without *Wolbachia* (strain *w*Mel) and *Aedes albopictus* cells (C710) with and without *Wolbachia* (strain *w*Stri) were grown at 24 °C in Shields and Sang M3 insect media (Sigma-Aldrich, Burlington, MA, USA) supplemented with 10% heat-inactivated fetal bovine serum (Gibco, Waltham, MA, USA), 1% each of l-glutamine (Corning, Corning, NY, USA), non-essential amino acids (Corning, Corning, NY, USA) and penicillin-streptomycin-antimycotic (Corning, Corning, NY, USA). Baby hamster kidney fibroblast (BHK-21) cells were grown at 37 °C under 5% CO_2_ in 1× Minimal Essential Medium (Corning, Corning, NY, USA) supplemented with 10% heat-inactivated fetal bovine serum (Corning, Corning, NY, USA), 1% each of L-glutamine (Corning, Corning, NY, USA), non-essential amino acids (Corning, Corning, NY, USA) and penicillin-streptomycin-antimycotic (Corning, Corning, NY, USA).

### 2.2. Fly Husbandry, Genetic Crosses and Virus Injections

The following stocks were obtained from the Bloomington *Drosophila* Stock Center (BDSC) located at Indiana University Bloomington (http://flystocks.bio.indiana.edu/, accessed on 2 March 2018). *Wolbachia*-infected RNAi mutant stock 60092 (y[1] sc[*] v[1] sev[21]; P{y[+t7.7] v[+t1.8]=TRiP.HMC05086}attP40) was used for shRNA-mediated knockdown of IPOD gene expression by driving dsRNA expression using previously described Act5C-Gal4 driver males (a generous gift from Dr. Brian Calvi; y^1^ w*; P{w[Act5C-GAL4}17bFO1/TM6B, Tb^1^). The homozygous TRiP mutant adult females colonized with *Wolbachia* were crossed to uninfected w; Sco/Cyo males. Virgin progeny females carrying the inducible shRNA construct were collected and age-matched (2–5 days old) before being crossed to the aforementioned Act5C-Gal4 driver males. As per our previous study, *Wolbachia*-infected TRiP mutant stock 42906 (y1 sc* v1; P {TRiP.HMS02599} attP40) was used to achieve knockdown of *Mt2* gene expression by driving dsRNA expression using the aforementioned Act5C-Gal4 driver males. All fly stocks were maintained on a standard cornmeal-agar medium diet supplemented with penicillin and streptomycin (P/S) at 25 °C on a 24 h light/dark cycle. To establish a systemic virus infection in vivo, flies were anesthetized with CO_2_ and injected intrathoracically with 50 nL of approximately 10^10^ PFU/mL of purified Sindbis virus (SINV-nLuc) (as in [6]) or sterile saline solution (1× PBS) using a nano-injector (Drummond Scientific, Broomall, PA, USA). Flies were collected two days post-infection, snap-frozen in liquid N_2_, and stored at −80 °C for downstream processing. Samples for quantitative PCR and quantitative RT-PCR were homogenized in TRiZOL reagent (Sigma Aldrich, Burlington, MA, USA) and further processed for nucleic acid extractions using the manufacturer’s protocols.

### 2.3. DNMT2 Overexpression in Insect Cells

Expression vectors containing *Drosophila melanogaster* and *Aedes albopictus* DNMT2 orthologs used here were designed in the following manner: *Aedes albopictus AMt2* coding region was subcloned into PCR 2.1 TOPO vector (Invitrogen, Waltham, MA, USA) by PCR amplification of cDNA generated from reverse-transcribed total cellular RNA isolated from C6/36 *Aedes albopictus* cells using Protoscript II RT (NEB) and oligo-dT primers (Integrated DNA Technologies, Coralville, IO, USA). The coding region was validated via sequencing before being cloned into the pAFW expression vector (1111) (Gateway Vector Resources, DGRC, Bloomington, IN, USA), downstream of and in-frame with the 3× FLAG tag using the native restriction sites AgeI and NheI (NEB, Ipswich, MA, USA). Expression of FLAG-tagged AaDNMT2 in C710 *Aedes albopictus* cells colonized with and without *Wolbachia* strain *w*Stri was confirmed using qRT-PCR and Western Blots using anti-FLAG monoclonal antibody (SAB4301135—Sigma-Aldrich, Burlington, MA, USA).

*Drosophila Mt2* (FBgn0028707) cDNA clone (GM14972) obtained from DGRC (https://dgrc.bio.indiana.edu/, accessed on 8 October 2017) was cloned into the pAFW expression vector (1111, https://dgrc.bio.indiana.edu/, accessed on 21 June 2017) with an engineered SaII site (Gateway Vector Resources, DGRC, Bloomington, IN, USA) downstream of and in-frame with the 3× FLAG tag using Gibson assembly (HiFi DNA assembly mix, NEB, Ipswich, MA, USA). Expression of FLAG-tagged DNMT2 in fly cells was confirmed using qRT-PCR and Western Blots using an anti-FLAG monoclonal antibody (SAB4301135—Sigma-Aldrich, Burlington, MA, USA). JW18 fly cells were transfected with expression constructs using Lipofectamine LTX supplemented with Plus reagent (Invitrogen, Waltham, MA, USA) by following the manufacturer’s protocols. Protein expression was assessed 72 h post-transfection via Western Blot using a monoclonal antibody against the FLAG epitope (Sigma, Burlington, MA, USA). Each Western blot experiment included cellular β-actin levels as loading controls probed using a monoclonal anti-β-actin antibody.

### 2.4. Virus Infection in Cells

Viral titers were determined using standard plaque assays on baby hamster kidney fibroblast (BHK-21) cells. Cells were fixed 48 h post-infection using 10% (*v*/*v*) formaldehyde and stained with crystal violet to visualize plaques.

### 2.5. Real-Time Quantitative PCR and RT-PCR Analyses

Total DNA and RNA were extracted from samples using TRiZOL reagent (Sigma Aldrich, Burlington, MA, USA) according to the manufacturer’s protocols. Synthesis of complementary DNA (cDNA) was carried out using MMuLV Reverse Transcriptase (NEB, Ipswich, MA, USA) and random hexamer primers (Integrated DNA Technologies, Coralville, IO, USA). Negative (no RT or no gDNA or cDNA synthesized from mock-infected cell supernatants) controls were used for each target per reaction. Quantitative PCR or RT-PCR analyses were performed using Brilliant III SYBR Green QPCR master mix (Bioline, Cincinnati, OH, USA) with gene-specific primers on an Applied Bioscience StepOnePlus qPCR machine (Life Technologies, Carlsbad, CA, USA). All primer sets were designed based on information present in existing literature [14,17,20]. Target gene expression levels were normalized to the endogenous 18S rRNA expression using the delta-delta comparative threshold method (ΔΔCT) (Table S1).

### 2.6. Phylogenetic Analyses

Multiple sequence alignments were generated using Clustal Omega. Tree topologies were obtained using RAxML with aligned codon-based nucleotide sequences. The “-m GTRGAMMA” model was used with rapid bootstrap analysis and search for the best tree (option: -f a), and 100 bootstrap replicates [26]. Final trees were visualized using FigTree v1.4.4.

### 2.7. CodeML Analyses

The codeML null and alternative branch-site models were run for each individual branch in the tree as foreground independently [27]. In the alternative model, the branch-site model allows a class of sites in the foreground branch to have a dN/dS > 1. In the text, we generally refer to dN/dS as ω. Convergence issues were addressed by rerunning analyses with different values for Small_Diff. Signs of convergence issues include (1) lnL values worse than the M1a NearlyNeutral site model; (2) the first two site classes having proportions of zero; (3) the null model having better lnL than the alternative model; (4) in the alternative model, lnL values worse than expected given estimated site posterior probabilities.

### 2.8. In Silico miRNA Prediction

Prediction of miRNAs targeting Drosophila Mt2 (FBgn0028707) and IPOD (FBgn0030187) was carried out using two independent miRNA prediction servers, TargetScanFly v7.2 and microrna.org [28,29]. The latter combines miRanda target prediction with an additional mirSVR target downregulation likelihood score [30]. Accession numbers of miRNAs predicted in this study were obtained from miRBase.

### 2.9. Protein Conservation

Protein conservation was determined with the Protein Residue Conservation Prediction tool (http://compbio.cs.princeton.edu/conservation/index.html, accessed on 30 October 2019) [31,32]. Multiple sequence alignment of amino acid sequences carried out using Clustal Omega was used as input. At the same time, Shannon entropy scores were selected as output, alongside a window size of zero, and sequence weighting was set to “false.” Conservation was subsequently plotted using GraphPad Prism 8. DNMT2 motif regions were defined as per described in previous studies [33]. For IPOD, domains were defined based on Pfam and InterPro domain prediction results obtained using *Drosophila melanogaster* IPOD as an input query [34,35].

### 2.10. Homology Modelling of DNMT2 Orthologs

Template-based comparative modeling of DNMT2 orthologs from *Drosophila melanogaster*, *Aedes albopictus*, and *Anopheles gambiae* was performed using the intensive modeling approach in Protein Homology/Analogy Recognition Engine 2 (Phyre2) [35]. Protein structures were visualized using PyMOL (The PyMOL Molecular Graphics System, Version 1.2r3pre. Schrödinger, LLC, New York, NY, USA).

### 2.11. Inter-Protein Co-Evolution Analyses

Co-evolution of Drosophila DNMT2 and IPOD orthologs was performed using multiple sequence alignments using the MirrorTree Server [36]. In addition, Robinson–Foulds distance was calculated to measure the dissimilarity between the topologies of unrooted IPOD and DNMT2 phylogenetic trees using the Visual TreeCmp web server [37,38]. The following optional parameters were selected for Weighted Robinson–Foulds, RFWeighted (0.5), and RF (0.5) analyses: normalized distances prune trees and zero weights allowed.

### 2.12. Statistical Analyses of Experimental Data

All statistical tests were conducted using GraphPad Prism 8 (GraphPad Software Inc., San Diego, CA, USA). Details of statistical tests for each experiment can be found in the results section and the associated figure legends.

### 2.13. Graphics

Graphical assets made in BioRender—biorender.com, accessed on 5 March 2020.

## 3. Results

### 3.1. AaDNMT2 and DmDNMT2 Differ in Structure

Both *Drosophila* and *Aedes* DNMT2 orthologs play essential roles in the tripartite interaction between *Wolbachia*-host–virus, which suggests the overall importance of this MTase in mediating host-pathogen interactions [6,17]. Notably, however, in contrast to the antiviral nature of *Drosophila* DNMT2 (hereafter referred to as *Dm*DNMT2), the effects of *Aedes aegypti* (hereafter referred to as *Ae*DNMT2) and *Aedes albopictus* (*Aa*DNMT2) DNMT2 orthologs are distinctly proviral [7]. Therefore, we wondered whether potential differences in structure and/or regulation exist that might contribute to the observed differences of these DNMT2 orthologs, reasoning that evidence of such differences, if present, could indicate distinct molecular evolution between MTase orthologs from members of Culicidae and Drosophilidae families.

**Figure 1:**
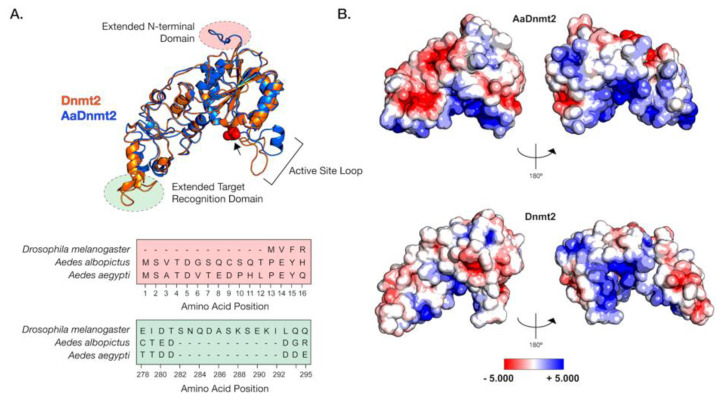
Structural differences between Drosophila and Aedes DNMT2 orthologs. Structures of DNMT2 orthologs from *Drosophila melanogaster* (*Dm*DNMT2) and *Aedes albopictus* (AaDNMT2) were generated using homology modeling (Phyre 2). (**A**) Superimposed ribbon diagrams of DNMT2 orthologs from *Drosophila melanogaster* (DNMT2, blue) and *Aedes albopictus* (AaDNMT2, in orange) outline key structural differences. Primary sequence alignment of the two orthologs (46% overall amino acid sequence identity) indicates significant differences in the N-terminal end (shown in pale red on the ribbon diagram and the sequence alignment below) and the target recognition domains (TRDs) (shown in pale green on the ribbon diagram). The catalytic cysteine residue (Cys 78) present within the highly conserved PPCQ motif is represented as red spheres). (**B**) Electrostatic potential surface visualization models of DNMT2 orthologs were generated through PyMOL 2.4 (Schrödinger, LLC.) using the in-built Adaptive Poisson–Boltzmann Solver (APBS) plug-in. Colored scale bars indicate the range of electrostatic potentials calculated based on amino acid compositions of each DNMT2 ortholog. The rotation symbol reflects structural features viewed 180° apart along the vertical axis.

First, we assessed the broader differences in protein sequence across members of Culicidae and Drosophilidae. Multiple sequence alignment of DNMT2 primary amino acid sequences indicate that differences between overall fly and mosquito DNMT2 orthologs are most notable in the N-terminal end and the C-terminal (residues 282–292) target recognition domains (TRDs), as evidenced by the lower degree of primary sequence conservation in these two regions. The N-terminal end of mosquito DNMT2 is variable in length across different taxa in the Culicidae family. It is, on average, 7–12 aa longer than the Drosophilidae counterparts, with the *Anopheles darlingi* DNMT2 ortholog being 47 aa longer in length (Figure S1). In contrast to mosquito DNMT2, we found only two instances of extended N-termini within Drosophilidae DNMT2: *Drosophila busckii* (17 aa) and *Drosophila serrata* (4 aa). We found an overall lack of N-terminal sequence conservation among the different Culicidae orthologs, aside from a few residues that are conserved within members of the *Aedes* and *Anopheles* genera. In silico prediction analyses also showed this region to be devoid of any ordered secondary structure, suggesting conformational flexibility and potential to participate in protein-protein interactions. The other prominent difference in primary sequence between DNMT2 orthologs from these two Dipteran families occurs within the TRD, extended (10–12 aa) in the vast majority of Drosophilidae DNMT2s, except for *Drosophila ananassae* and *Drosophila bipectinata* (Figure S1). However, unlike the N-terminal extension within Culicidae, the extended TRD of Drosophilidae DNMT2 contains a conserved stretch of three residues (KSE) that constitutes the start of a predicted α-helix (Figure 1, Figure S1). Taken together, it is conceivable that such differences in the TRD contribute to differential substrate–MTase interactions between Culicidae and Drosophilidae DNMT2 orthologs.

In light of these structural differences between mosquito and *Drosophila* orthologs, we focused on the *Aedes albopictus* and *Drosophila melanogaster* proteins (*Aa*DNMT2 (344 aa) and *Dm*DNMT2 (345 aa), respectively) as these are model organisms where we could mechanistically compare their function and cellular context. The *Aa*DNMT2 and *Dm*DNMT2 orthologs are comparable in size, sharing 46% amino acid sequence identity. However, as shown above, these orthologs exhibit significant differences in two regions: the N-terminus and the TRD (Figure 1, Figure S1). We, therefore, compared their tertiary structures to identify how these differences might affect their respective structures. The extended N-terminal end of *Aa*DNMT2 remained surface exposed in an unstructured, flexible conformation, allowing for contact with potential interaction partners (Figure 1A). The extended TRD region within *Dm*DNMT2 was also found to be mostly surface exposed, adopting a short α-helical conformation at the C-terminal end. Comparison to crystal structures of DNMT2 from armyworm (*Spodoptera frugiperda, PDB ID: 4HON*) and fission yeast (*Schizosaccharomyces pombe*, *PDB ID: 6FDF*) indicate that the rest of the TRD is unstructured and conformationally flexible. Given the possible importance of the conformational state of this TRD region for interactions with the nucleic acid substrate, the extended region within *Dm*DNMT2 carries the potential to alter substrate-MTase interactions [39].

Outside of the two aforementioned regions, other notable structural differences when comparing the *D. melanogaster* and *A. albopictus* homologs are in the 20 aa in length active site loop region adjacent to the catalytic PPCQ motif. This region appears more structured in *Aa*DNMT2 relative to *Dm*DNMT2, consisting of a short stretch of residues forming an α-helix (Figure 1A). We found this feature to be consistent with the in silico secondary structure prediction for this *Aa*DNMT2 region. However, in contrast to the estimated 3D structure, this α-helical stretch was predicted to be extended for *Dm*DNMT2, spanning the entirety of the active site loop. This is likely a result of differences in the amino acid composition within this region between the two orthologs, where residues present within *Aa*DNMT2, e.g., Proline (P), Valine (V), Phenylalanine (F), are more likely to disrupt the formation of α-helices. However, this region has been suggested to adopt different structural conformations, switching between structured and unstructured α-helices, upon nucleic acid binding [39]. Multiple sequence alignment and structural modeling of Culicidae and Drosophilidae DNMT2 orthologs suggests that this feature is consistent within members of the respective families. Additionally, these modeled structures are built on crystal snapshots of otherwise dynamic protein structures, limiting our interpretation given that each structure is constrained to a particular, static conformation.

Aside from differences in secondary and tertiary structure, physicochemical properties of amino acids contribute to their spatial distribution and the propensity to remain either buried or exposed in a solvent-accessible conformation. This attribute of proteins can also influence interactions with other biomolecules, which for enzymes such as MTases include cognate interaction partners such as regulators or nucleic acid substrates. We, therefore, asked whether *Aa*DNMT2 and *Dm*DNMT2 differ significantly in terms of their surface charge distribution profiles. Mapping of electrostatic charge densities on solvent-accessible 3D surfaces revealed an overall greater distribution of charged residues on the surface of *Aa*DNMT2. This included a distinctly larger patch of negatively charged residues in the TRD (Figure 1B). As expected, both DNMT2 orthologs contained a high density of positive charge in and around the catalytic region known to bind the negatively charged nucleic acid substrate. Additionally, in line with its role in substrate binding, the catalytic helix adjacent region of *Aa*DNMT2 was determined to be largely positively charged. This attribute, however, was noticeably absent from *Dm*DNMT2, whose extended catalytic helix adjacent region was found to be moderately negatively charged (Figure 1B).

Taken together, although the structural superposition of *Aa*DNMT2 and *Dm*DNMT2 demonstrates overall structural congruency between the two orthologs, significant differences remain, which potentially indicate unique protein-protein or protein–substrate interactions for each ortholog.

### 3.2. Drosophila IPOD Regulates DmDNMT2 Expression

Pathways and host factors involved in regulating DNMT2 expression in dipteran insects are poorly understood. In a past study, a potential host factor in *Drosophila melanogaster*, the aptly named interaction partner of DNMT2 or IPOD, was shown to regulate *Dm*DNMT2 expression and function [40]. However, it is unclear whether IPOD is involved in DNMT2 regulation within all Dipteran insects or whether distinct modes of DNMT2 regulation have evolved across different Dipteran families. In light of our previous results highlighting differences between Drosophilidae and Culicidae DNMT2, we investigated the presence and conservation of IPOD orthologs among the species included in this study. Additionally, we examined the role of this protein in DNMT2 regulation within *Drosophila melanogaster*.

**Figure 2:**
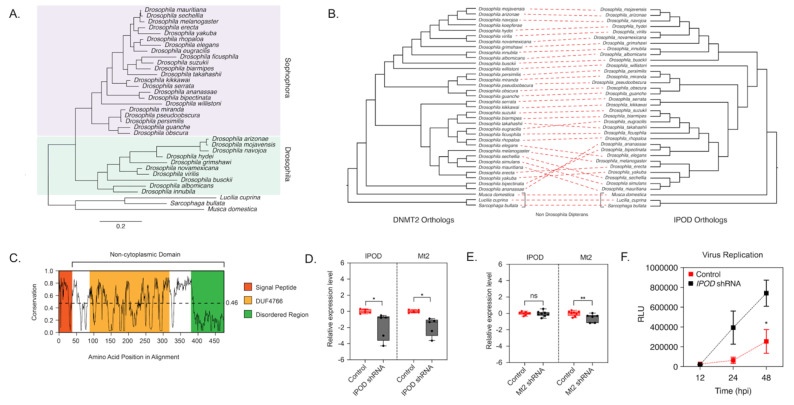
IPOD regulates DNMT2 expression in Drosophila. (**A**) maximum-likelihood [43] tree of the interaction partner of DNMT2 (*IPOD*) gene present in multiple *Drosophila* species was constructed using RAxML using a multiple sequence alignment of *IPOD* nucleotide sequences. The sequence of the *IPOD* orthologs from *Lucilia cuprina*, *Musca domestica*, and *Sarcophaga bullata* were used as outgroups. Scale bars represent branch lengths. (**B**) Inter-protein co-evolutionary analyses of DNMT2 and IPOD orthologs were performed using the TreeCmp software packages. Red dashed lines connect the same Drosophila taxon. (**C**) Shannon conservation plot representing the degree of conservation (*Y*-axis) of IPOD orthologs present at every amino acid position (*X*-axis) across *Drosophilids*. The horizontal dashed line indicates the mean conservation score (0.46) across all amino acid positions. Colored boxes represent three InterPro domains identified across all IPOD orthologs in *Drosophilids*, including the N-terminal signal peptide (depicted in orange), followed by a C-terminal non-cytoplasmic domain (depicted in white) consisting of a conserved domain of unknown function (DUF4766, depicted in yellow) and a glycine-rich disordered region (depicted in green) present at the C-terminal end. (**D**,**E**) *IPOD* is an upstream regulator of *Mt2* expression in *Drosophila melanogaster*. (**D**) *IPOD* expression was knocked down in *Wolbachia w*Mel-colonized *Drosophila melanogaster* (TRiP line# 60092) by driving expression of a targeting short-hairpin RNA (shRNA) against the target IPOD mRNA. Relative expression of the target *IPOD* mRNA and *Mt2* mRNA was assessed via quantitative RT-PCR using total RNA derived from age-matched females. Siblings lacking the shRNA were used as the negative control. Two-tailed *t*-tests on log-transformed values; *IPOD*: *p* < 0.05, t = 3.678, df = 8.00, *Mt2*: *p* < 0.05, t = 2.454, df = 8.00. Error bars represent the standard error of the mean (SEM) of experimental replicates (*n* = 5). (**E**) *Mt2* expression was knocked down in *Wolbachia w*Mel-colonized *Drosophila melanogaster* by driving expression of a targeting short-hairpin RNA (shRNA) against the target mRNA. Relative expression of the target *Mt2* mRNA and *IPOD* mRNA was assessed via quantitative RT-PCR using total RNA derived from age-matched females. Siblings lacking the shRNA were used as the negative control. Two-tailed *t*-tests on log-transformed values; *Mt2*: *p* < 0.01, t = 2.576, df = 12.00, *IPOD*: *p* = 0.717969, t = 0.3686, df = 14.00. Error bars represent the standard error of the mean (SEM) of experimental replicates (*n* = 6–8). (**F**) Effect of *IPOD* knockdown on *Wolbachia-*mediated virus inhibition. Age-matched *Wolbachia-*colonized female flies, either wild type or expressing *IPOD*-targeting shRNA, were intrathoracically injected with the SINV-nLuc virus. At indicated times post-infection (*X*-axis), flies were harvested and snap-frozen before homogenization. Homogenized lysates were used to measure luciferase expression (RLU, *Y*-axis), which was subsequently used as a proxy to quantify virus replication. Two-way ANOVA of multivariate comparisons with Sidak’s post hoc test; *IPOD* knockdown: *p* < 0.01, Time: *p* < 0.01. Error bars represent the standard error of the mean (SEM) of experimental replicates (*n* = 3/time point). * *p* < 0.05, ** *p* < 0.01, ns = not-significant.

A Protein-BLAST search of *Drosophila melanogaster* IPOD revealed IPOD orthologs among Dipteran insects that were also found to encode DNMT2 orthologs, but these were predominantly restricted to *Drosophila* species (Figure 2A, Figure S2). Importantly, this was not due to the absence of available sequence information, as nearly complete genome assemblies are present for all taxa except *Polypedilum vanderplanki*. Phylogenetic analysis of these *Drosophila* IPOD sequences revealed occurrence within both Drosophila and Sophophora subgroups of the Drosophila genus, with an average 46% amino acid sequence identity across all positions (Figure 2A,B). Furthermore, MirrorTree analyses of Drosophila DNMT2 and IPOD orthologs revealed significant mirroring of the branch lengths in the two phylogenies, consistent with a strong inter-protein co-evolutionary relationship between the two; correlation: 0.787, *p*-value ≤ 0.000001 (Figure 2). This was further validated by the results from TreeCmp analyses assessing the Robinson–Foulds (RF) and Matching Split [41] distances between Drosophila IPOD and DNMT2 trees, which showed a similar high congruence between the two tree topologies: RF (0.5) = 8, MS = 27.0. In contrast, very low congruence, with normalized distances ≤ 0.4 was found when the trees were compared to random trees generated according to Yule (RF/MS to YuleAvg) and uniform (RF/MS to UnifAvg) models; RF (0.5)_to UnifAvg = 0.3841, RF (0.5) to YuleAvg = 0.3852, MS_to UnifAvg = 0.2426, MS to YuleAvg = 0.2880. Domain analyses using Pfam and InterPro identified a DUF4766 (PF15973) domain (residues 82–232) present in all orthologs, and that 90% of the protein (residues 33–349) contains a non-cytoplasmic domain, with a smaller signal peptide domain (residues 1–32) present at the N-terminal end (posterior probability score > 0.99) [39,42] (Figure 2C). Notably, we found nearly 28% (97/397) of the total protein length to be made of glycine residues, associated with a high degree of disordered structure. Indeed, an IUPred search predicted large stretches of intrinsically disordered regions along the entire length of the protein (disorder tendency score > 0.5), indicating a potential role of IPOD in mediating complex protein-protein interactions [31,32]. Taken together, these features are consistent with IPOD’s role as a nuclear protein with a potential role in transcriptional regulation. Interestingly, IPOD lacks a canonical DNA-binding domain. Therefore, if IPOD regulates DmDNMT2 expression, it likely does so by interacting with other DNA-binding proteins.

Mt2 is not expressed in *Wolbachia-*free adult flies. However, when *Wolbachia* is present, we previously observed dramatic upregulation of the gene [6]. We have previously shown that this upregulation is important for *Wolbachia*-based antiviral protection [6]. To validate IPOD’s role in *Dm*DNMT2 regulation, we, therefore, used *Wolbachia-*infected flies, where DmDNMT2 expression is detectable. We used RNAi to knock down *IPOD* (IPOD) expression in vivo in a transgenic fruit fly model (TRiP stock# 60092) by driving the expression of IPOD targeting short-hairpin RNA (shRNA) and measured relative mRNA levels of both *IPOD* and *Mt2* genes. We also measured these levels within the context of transgenic RNAi flies (TRiP stock# 42906) expressing shRNA against *Dm*DNMT2 to determine whether knockdown *of Mt2* affected levels of IPOD transcripts. Indeed, as expected, knocking down *IPOD* expression led to significantly reduced *Mt2* mRNA levels in *Wolbachia* infected flies expressing *IPOD*-targeting shRNA (TRiP stock# 60092); two-tailed *t*-tests on log-transformed values; *IPOD*: *p* < 0.05, t = 3.678, df = 8.00, *Mt2*: *p* < 0.05, t = 2.454, df = 8.00 (Figure 2D). Conversely, depleting *Dm*DNMT2 (TRiP stock# 42906) did not cause any significant change in *IPOD* mRNA levels, suggesting that *IPOD* likely functions upstream in the regulatory pathway; *t*-tests on log-transformed values, *IPOD*, *Mt2*: *p* < 0.01, t = 2.576, df = 12.00, *IPOD*: *p* = 0.717969, t = 0.3686, df = 14.00 (Figure 2E).

Additionally, we wondered whether knockdown of IPOD affects virus inhibition within the context of a *Wolbachia-*colonized fly host. We reasoned that if IPOD is a positive regulator of *Dm*DNMT2 expression, and its loss led to a subsequent reduction in *Dm*DNMT2 levels, this may rescue the virus from *Wolbachia-*mediated inhibition, phenocopying our previous results [6]. Flies expressing *IPOD-*targeting shRNA were challenged with a SINV expressing a translationally fused luciferase reporter (SINV-nLuc) and virus replication at 12, 24, and 48 h post-infection was measured by quantifying luciferase activity as a proxy for viral gene expression. Knockdown of *IPOD* in *Wolbachia-*colonized flies led to a significant increase in viral RNA, likely due to reduced *Dm*DNMT2 levels; two-way ANOVA with Sidak’s post hoc multiple comparisons test; *IPOD* knockdown: *p* < 0.01, time: *p* < 0.01 (Figure 2F). Importantly, we controlled for *Wolbachia* infection levels to make sure that the virus replication increase we were seeing was not due to a loss in *Wolbachia* infection; unpaired Welch’s *t*-test: *p* = 0.4788, t = 0.7695, df = 4 (Figure S3). Collectively, these results support IPOD’s role in regulating *Dm*DNMT2 expression in the fruit fly.

### 3.3. Evidence of Adaptive Evolution in DNMT2

**Figure 3:**
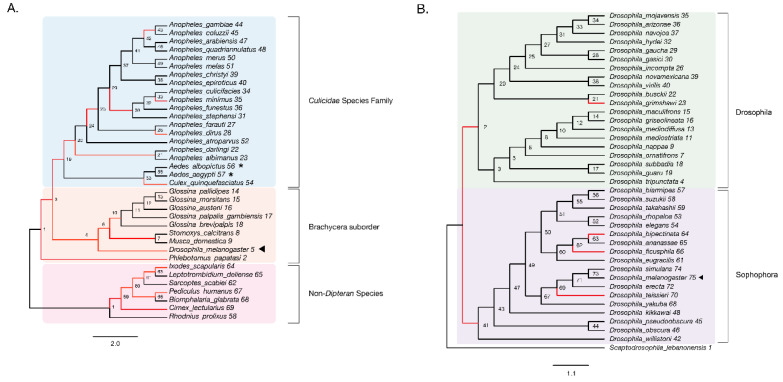
Evidence of adaptive evolution in DNMT2 orthologs. Branches numbered for reference in main text and Table 1. (**A**) branch-site tests were conducted to detect positive selection (ω > 1) across lineages of DNMT2 orthologs belonging to different species within the order Diptera (clades highlighted in light blue and orange for Culicidae family and the Brachycera suborder, respectively) and non-Dipteran (clades highlighted in light pink) animals. Branches with raw *p*-value < 0.05 for ω > 1 are represented in red. Filled left arrow = antiviral; * = proviral (**A**) Significant evidence (see Table 1 for details) of positive selection in DNMT2 is present along branches representing multiple insect species. These include several *Anopheles* and one *Culex* mosquito species, as well as several other Dipteran fly species. (**B**) Significant evidence of positive selection is present along the ancestral branch leading to the subgroup *Sophophora* (clades highlighted in light purple) and along branches leading to four *Drosophila* species.

Prior studies have demonstrated that a high proportion of amino acid changes in *Drosophila* are driven by positive selection, and although statistical problems with models used to estimate positive selection may lead to false positives, many studies, using different approaches, have detected a large proportion of positively selected sites in the *Drosophila* lineage, especially genes encoding for proteins that interact with pathogens [24,42,43,44]. For example, Sawyer et al. found that most (93%) of replacements present among 56 loci across *Drosophila melanogaster* and *Drosophila simulans* were beneficial. Given the evidence above regarding structural and regulatory differences in Mt2 homologs across fruit flies and mosquitos, as well as recent evidence of DNMT2′s role in RNA virus regulation, we wondered whether there is any evidence of positive selection in this gene, as had been reported in a smaller study, focused on *Drosophila* [45]. Here, we included DNMT2 orthologs from a total of 29 *Dipteran* insect species, which we evaluated for positive selection by maximum likelihood analyses using the branch-site model in CodeML (PAML package) [27]. Given the relevance of mosquitoes as disease vectors for viruses and other pathogens, our list included DNMT2 orthologs from a total of 20 species from the Culicidae family (Suborder: Nematocera), including 17 *Anopheles*, 2 *Aedes*, and 1 *Culex* species (Figure 3A). Additionally, we included DNMT2 orthologs from seven representative taxa spanning the Suborder Brachycera, including five members of the *Glossina* genus and one each from the following five genera: *Stomoxys*, *Musca, Drosophila*, and *Phlebotomus*. Finally, DNMT2 orthologs from six non-dipteran insects were included as outgroups (Figure 3A).

We found significant signatures of positive selection along the ancestral branches leading to Dipteran insects (Branches 2, 3) and the Culicidae (Branch 19), as well as along relatively recent branches within the Culicidae, with the notable exception of *Aedes* species (Branches 53, 55–57). Additionally, signatures were detected along the *Culex quinquefasciatus* lineage (*p* = 8 × 10^−6^, Branch 54) and several *Anopheles* species or recently diverged internal branches: *Anopheles dirus*, *Anopheles minimus*, and branches 21, 30, and 42 (Figure 3A). Additionally, we found instances of positive selection within deeper branches in the mosquito clade: branches 19, 20, and 25 (Figure 3A, Table 1). Outside of the Culicidae family, signatures of positive selection were detected along lineages within the Brachycera Suborder of Dipteran insects (Figure 3A, Table 1). These included all ancestral lineages leading to genera within this Suborder, representing members of *Glossina* species, *Musca domestica, Stomoxys calcitrans*, *Phlebotomus*, and importantly, *Drosophila melanogaster* (Branches 2, 4, 5, 6, 7 and 10, Figure 3A). Finally, the branch directly leading to *Drosophila melanogaster* was found to be under positive selection (Branch 5, Figure 3A). Taken together, these findings suggest an ongoing process of adaptive evolution in Dipteran DNMT2, suggesting the potential roles of several, yet uncharacterized, DNMT2 orthologs in host-pathogen interactions.

We next performed a finer-grained analysis of *Drosophila* DNMT2 orthologs across 38 different *Drosophila* species encompassing the *Sophophora* (20 species) and *Drosophila* (18 species) subgenera using the same branch-site method as above. The DNMT2 sequence from *Scaptodrosophila lebanonensis* (Scaptodrosophila Genus) was used as an outgroup. The phylogenetic tree of *Drosophila* DNMT2 orthologs inferred using maximum likelihood analyses was largely congruent with the previously reported phylogeny of *Drosophila* species, with distinct separation of DNMT2 orthologs into two known *Drosophila* subgroups (Figure 3B, Table 2) [46]. Strong evidence of positive selection (raw *p*-value = 0.002) was found in the lineage directly ancestral to all *Sophophora* (Branch 41) and weaker evidence (raw *p*-value = 0.027) for the ancestral lineage to all *Drosophila* (Branch 2) and the lineages leading to *Drosophila grimshawi* (Branch 23), *Drosophila bipectinata* (Branch 64), *Drosophila fiscusphila* (Branch 66), and *Drosophila teissieri* (Branch 70). Notably, positive selection was not found in *Drosophila melanogaster* (Branch 75), suggesting the absence of detectable adaptations since its divergence from other members of the *Sophophora* genus. These findings suggest several instances of recent adaptive evolution within *Drosophila* DNMT2 since its divergence from Culicidae. Notably, these results are in line with the findings reported by Vieira et al. [45].

### 3.4. Identification of Codon Sites under Positive Selection in DNMT2

The results from the above CodeML analyses suggested multiple instances of positive selection along Dipteran lineages. To identify codon sites having likely experienced positive selection, we used the Bayes Empirical Bayes (BEB) posterior probabilities from CodeML to identify amino acid sites having experienced positive selection (ω > 1) within the protein-coding regions of DNMT2. Notably, we found several sites from the ω > 1 class with >95% probability across multiple Dipteran lineages (Table 1) and, more specifically, within the *Drosophila* genus (Table 2). Given their differential roles in host immunity relative to *Drosophila* DNMT2, we chose to focus our attention on codon sites present within lineages ancestral to or leading to *Aedes albopictus*.

It is possible for changes identified along internal branches to have changed again later in some lineages. We, therefore, looked at sites identified on internal branches to see which extant taxa still have them by assessing the degree of conservation at these sites within Culicidae and Drosophilidae families (Figure S5). Two sites (44G, 55S) identified as being under selection among all Dipteran DNMT2s (Branch 3, Table 1) were found to be conserved in >80% of Culicidae and Drosophilidae species. Of the two sites, the variant 44G was conserved in most taxa (>83%, 24/30). In contrast, a conserved replacement site (55S) was found in the vast majority of species (>97%, 29/30), with only one *Anopheles* species harboring a G at site 55. Thus, aside from a few exceptions, conservation of the codon sites identified within Culicidae and Drosophilidae were limited to taxa within these respective families. Within Culicidae, our BEB analyses identified 19 amino acid positions under selection (Branches 54,19–21,28,30, Table 1). Mapping these sites on a multiple sequence alignment of Culicidae species identified four amino acid sites unique to a single species. In contrast, the rest of the amino acid residues under selection were present among multiple Culicidae taxa (Figure S5A). Notably, despite the absence of recent selection along the *Aedes* lineage, nine ancestral changes (Branch 19, Table 1) are maintained in extant *Aedes* DNMT2 sequences.

We next performed BEB analysis to identify codon sites under selection within *Drosophila* DNMT2. To represent all adaptive amino acid changes that have occurred in this taxa over its entire evolutionary period, four sites explicitly identified in *Drosophila melanogaster* (Figure 3A, Branch 5, Table 1) were grouped alongside those identified in the most ancestral (2 sites) and most recent (2 sites) Dipteran lineages (Figure 3A Branches 3 and 4, Table 1), as well as sites identified in our *Drosophila-*specific analyses (3 sites) appearing on the ancestral lineage to the Sophophora subgenus (Figure 3B, Table 2). Mapping these 11 sites identified along lineages ancestral to *Drosophila melanogaster* revealed near-perfect conservation within Drosophila species from both Sophophora and Drosophila, suggesting that these changes occurred before the divergence of these subgroups. In contrast, sites identified along the branch ancestral to Sophophora were restricted to members of this subgroup (Figure S5B). It should be noted that these three Sophophora-specific codon sites agree with those identified by Vieira et al. (Table 2) [45]. In addition, none of the nine replacement amino acids unique to Drosophila were identified at the corresponding sites within members of the Culicidae, with one exception (Figure S5B, Table 1 and Table 2).

**Figure 4:**
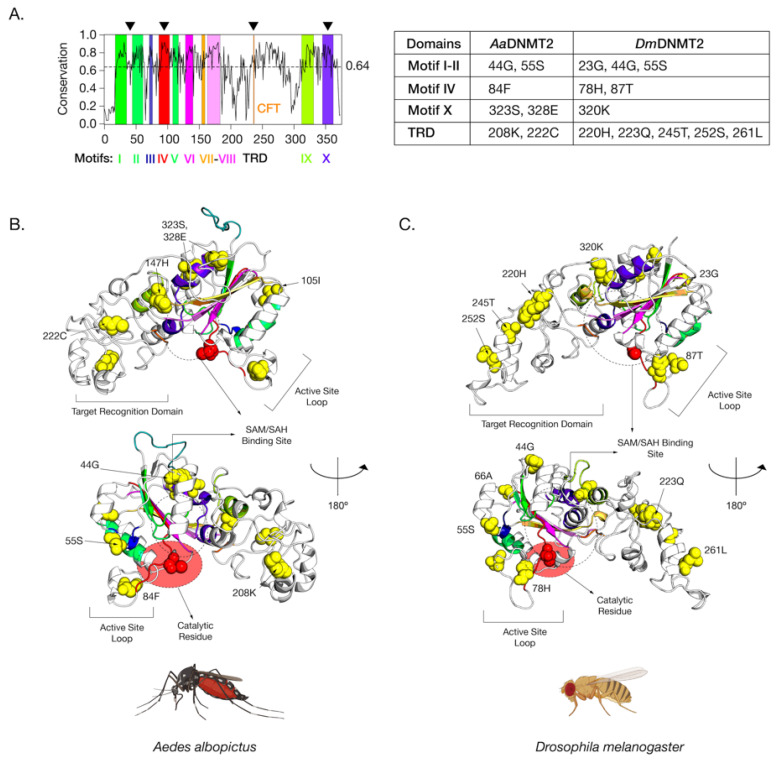
Amino acid positions in *Drosophila melanogaster* DNMT2 potentially under positive selection. (**A**) Shannon conservation plot representing the degree of conservation (*Y*-axis) of DNMT2 orthologs present at every amino acid position (*X*-axis) across within DNMT2 orthologs from mosquitoes and fruit flies. Colored boxes represent known DNMT2 functional motifs and domains involved in catalytic activity and target recognition (CFT). The mean conservation score (64%) across all amino acid positions is represented by the horizontal dotted line. Black arrows present on the top represent four major regions containing most amino acid positions with evidence of positive selection and high posterior probability values (>95%). These individual amino acids are also represented in the accompanying table to the right. (**B**,**C**) Spatial distribution of sites unique to each family is represented as yellow spheres on ribbon models of (**B**) *Aedes albopictus* (9 sites) and (**C**) *Drosophila melanogaster* (12 sites) DNMT2 structures visualized in PyMOL 2.4 (Schrödinger, LLC). The catalytically active cysteine residue (Cys, **C**) is represented in red. The predicted substrate, i.e., S-adenosyl methionine (SAM) or S-adenosyl homocysteine (SAH), the binding region is shown as a dashed oval. Functionally important active site loop and target recognition domains are also indicated on each structure. The lower structures are rotated 180° relative to the upper ones around the vertical axis.

We next mapped these identified sites on the primary amino acid sequence of DNMT2 to determine their locations relative to previously identified functionally important regions [33]. Eukaryotic DNMT2 is broadly divided into two domains, the catalytic domain, and the TRD. The former can be further divided into ten functional motif regions (I–X) (Figure 4). Analyses of amino acid conservation across all sites between DNMT2 orthologs from Drosophila and Culicidae families suggest an overall 64% conservation in the primary amino acid sequence, with a higher degree of conservation 77% within the catalytic region and 56% for the rest (Figure S5). Of the nine *Aedes* sites, whose ancestral branches (Figure 3, branches 3 and 19) showed >95% posterior probability identified from the BEB analyses, five were present within the catalytic domain (Figure S5). These include one (55S) in the Motif II region, one (84F) in the active site loop adjacent to the catalytic PPCQ Motif IV region, and two (323 S, 328 E) within the final Motif X region. One additional site (105I) was present within the catalytic domain, albeit in a non-motif region. The rest of the four identified sites were mapped to the TRD (Figure S5A). We next plotted the 11 sites from branches leading to D. melanogaster with >95% posterior probability identified from our BEB analyses along the primary *Dm*DNMT2 amino acid sequence (Figure S5B). Although it failed to reach the 95% posterior probability cutoff, a twelfth site 66A (BEB posterior probability > 90%) could be of interest and was included in our structure mapping analyses. It is almost completely restricted to Drosophilidae DNMT2 and is located within the catalytic region (Figure S5B).

While mapping the locations on the primary sequence allowed us to gauge the general location and conservation of these sites on the DNMT2 proteins of Culicidae and Drosophilidae to assess the spatial importance of the amino acid sites identified in our BEB analyses with respect to MTase function, we next mapped a subset of the sites that were found within *Aedes albopictus* and *Drosophila melanogaster* on the 3D structures of *Aa*DNMT2 and *Dm*DNMT2, respectively (Figure 4). These orthologs were chosen as representative members of the Culicidae and Drosophilidae families, given their previously described roles in virus regulation and host immunity [6,7,17,23]. Due to the absence of empirical structural information regarding *Aa*DNMT2 and *Dm*DNMT2, an intensive structural modeling approach using existing, experimentally solved crystal structures gathered from the Protein Data Bank (PDB) was used to generate predicted structures of these two DNMT2 orthologs (Figure 4). Furthermore, to better understand the spatial distribution of the sites relative to the canonical MTase catalytic binding pocket, we used molecular docking to introduce the methylation substrate *S*-adenosyl-*L*-homocysteine (SAH) to identify the co-factor binding pocket [47].

The positively selected sites across both the AaDNMT2 and DmDNMT2 structure map to the same structural regions in the protein (Figure 4A). These regions include (1) region-spanning Catalytic Motifs I and II (*Aa*DNMT2: 2 sites, 44G, 55S; *Dm*DNMT2: 3 sites, 23G, 44G, 55S), (2) Catalytic Motif IV Region and adjacent “active site loop” (*Aa*DNMT2: 1 site, 84F; *Dm*DNMT2: 2 sites, 78H, 87T), (3) Catalytic Motif X Region adjacent to the binding pocket for the canonical MTase co-factor *S*-adenosyl-methionine SAM and its resulting product *S*-adenosyl-homocysteine SAH (*Aa*DNMT2: 2 sites, 323S, 328E; *Dm*DNMT2: 1 site, 320K), (4) TRD involved in interactions with the nucleic acid target, facing away from the binding pocket, flanking the conserved CFT motif (*Aa*DNMT2: 2 sites, 208K, 222C; *Dm*DNMT2: 5 sites, 220H, 223Q, 245T, 252S, 261L) (Figure 4A). Notably, past studies indicate that these four regions contribute significantly towards DNMT2′s MTase activity with regard to substrate binding and catalytic activity [40]. Furthermore, high clustering of sites in the TRD region is significant, given that they (*Aa*DNMT2: 208, *Dm*DNMT2: 261L) are located in the catalytically critical region that is known to penetrate the major groove of the nucleic acid substrate [41]. Finally, for both orthologs, a large proportion of sites present at the N-terminus (*Aa*DNMT2: 44G,55S,105I, *Dm*DNMT2: 23G,44G,55S,66A) and the TRD (*Aa*DNMT2: 147H, 222C, *Dm*DNMT2: 220H, 223Q, 245T, 252S) were found to be present on the solvent-accessible surface (Figure 4B,C). These observations are in line with prior evidence suggesting that solvent exposure of protein surfaces has the most substantial impact on adaptive mutations, likely driven by unique intermolecular interactions [48]. Indeed, we found this feature not limited just to *Aa*DNMT2 and *Dm*DNMT2, as mapping the positively selected sites on the tertiary structure of *Anopheles darlingi* DNMT2 revealed a vast majority of sites to occur on the solvent-accessible protein surface (Figure 1B,C). Taken together, these observations suggest potential functional consequences of these amino acid substitutions on *Aedes albopictus* and *Drosophila melanogaster* DNMT2 with regard to catalytic activity or protein-protein interactions.

### 3.5. Antiviral Role of DmDNMT2 Is Host Dependent

As noted above, mosquito *Mt2* is proviral [7], while in *Drosophila*, *Mt2* is antiviral [6]. This difference could be due to the significant structural differences and divergent selection we noted above, differences in the host cellular environment for these two orthologs, or both. Therefore, we attempted to ask whether the antiviral role of these orthologs was dependent on the host cellular environment by carrying out heterologous expression of *Dm*DNMT2 and *Aa*DNMT2 in their non-native *Aedes albopictus* and *Drosophila melanogaster* cells alongside their native counterparts. We then assessed the effect of this heterologous expression on virus replication. We recognize the limitations of our approach given that these insect cells contain both their native *Mt2* and the heterologously expressed form. However, given the low levels of native DNMT2 expression in the cell, we reasoned that ectopic expression of either native or non-native ortholog should allow it to function as the dominant MTase variant. Additionally, we cleared the cell lines of *Wolbachia* infection to examine the role of this important variable, which could influence *Mt2* function.

**Figure 5:**
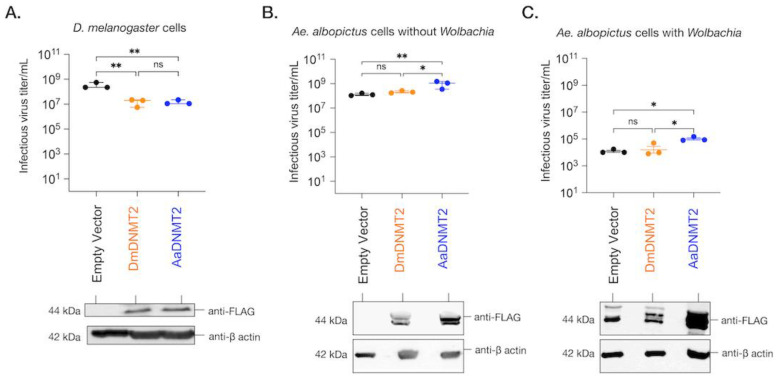
The effect of DNMT2 orthologs on virus replication is host-dependent. (**A**) *Drosophila melanogaster*-derived JW18 cells (without *Wolbachia*) were transfected with plasmid constructs expressing epitope (FLAG)-tagged versions of either the native fly (*Dm*DNMT2, depicted in orange) or the non-native mosquito (*Aa*DNMT2, depicted in blue) orthologs. Empty vector carrying only the FLAG tag was used as a negative control (depicted in black). At 72 h post-transfection, JW18 cells expressing either the empty vector, the native DNMT2 (*Dm*DNMT2), or the non-native DNMT2 (*Aa*DNMT2) were challenged with SINV at MOI of 10 particles/cell. Cell supernatants were collected 48 h post-infection, and infectious virus production was assessed via standard plaque assays on mammalian fibroblast BHK-21 cells. One-way ANOVA with Tukey’s post hoc test for multiple comparisons: empty vector vs. *Dm*DNMT2: *p* = 0.0016, empty vector vs. *Aa*DNMT2: *p* = 0.0017, *Dm*DNMT2 vs. *Aa*DNMT2: *p* = 0.9971. Error bars represent the standard error of the mean of three independent experiments. (**B**) *Aedes albopictus*-derived C710 cells (without *Wolbachia*) were transfected with plasmid constructs expressing epitope (FLAG)-tagged versions of either the native fly (*Dm*DNMT2, depicted in orange) or the non-native mosquito (*Aa*DNMT2, depicted in blue) orthologs. Empty vector carrying only the FLAG tag was used as a negative control (depicted in black). At 72 h post-transfection, cells were challenged with SINV at MOI of 10 particles/cell. Cell supernatants were collected 48 h post-infection, and infectious virus production was assessed via standard plaque assays on mammalian fibroblast BHK-21 cells. One-way ANOVA with Tukey’s post hoc test for multiple comparisons: empty vector vs. *Dm*DNMT2: *p* = 0.4788, empty vector vs. *Aa*DNMT2: *p* < 0.01, *Dm*DNMT2 vs. *Aa*DNMT2: *p* < 0.05. Error bars represent the standard error of the mean of three independent experiments. (**C**) *Aedes albopictus*-derived C710 cells (with *Wolbachia* strain *w*Stri) were transfected with plasmid constructs expressing epitope (FLAG)-tagged versions of either the native fly (*Dm*DNMT2, depicted in orange) or the non-native mosquito (*Aa*DNMT2, depicted in blue) orthologs. Empty vector carrying only the FLAG tag was used as a negative control (depicted in black). At 72 h post-transfection, cells were challenged with SINV at MOI of 10 particles/cell. Cell supernatants were collected 48 h post-infection, and infectious virus production was assessed via standard plaque assays on mammalian fibroblast BHK-21 cells. One-way ANOVA with Tukey’s post hoc test for multiple comparisons: empty vector vs. *Dm*DNMT2: *p* = 0.8709, empty vector vs. *Aa*DNMT2: *p* < 0.05, *Dm*DNMT2 vs. *Aa*DNMT2: *p* < 0.05. Error bars represent the standard error of the mean of three independent experiments. ** *p* < 0.01, * *p* < 0.05, ns = not-significant. DNMT2 protein expression was assessed 72 h post-transfection via Western Blot using antibodies against the FLAG-epitope in all three cases. Cellular β-actin protein expression, probed using an anti-β-actin antibody, was used as a loading control.

Previous work has demonstrated that ectopic expression of *Dm*DNMT2 in *Drosophila melanogaster-*derived JW18 cells causes a reduction in infectious virus production, mirroring its antiviral role in vivo [6,17]. Altogether, *Dm*DNMT2 can restrict multiple viruses from at least four distinct RNA virus families, highlighting a broad spectrum of antiviral activity. To determine whether this property is unique to *Dm*DNMT2 in the *Drosophila melanogaster* host, we expressed *Aa*DNMT2 in fly cells and tested its effect on infectious virus production following challenge with SINV. *Drosophila melanogaster-*derived JW18 cells (cleared of *Wolbachia* infection) were transfected with FLAG-tagged versions of *Dm*DNMT2 or *Aa*DNMT2 and were challenged with SINV at an MOI of 10 particles/cell approximately 72 h post-transfection. Cell supernatants were collected after 48 h post-infection, and viral titers were assayed on vertebrate baby hamster kidney fibroblast (BHK-21) cells using standard plaque assays. We saw a significant reduction in viral titer in cells expressing *Dm*DNMT2 compared to cells expressing the empty vector control. Notably, this result was phenocopied in cells expressing the non-native *Aa*DNMT2 ortholog; one-way ANOVA with Tukey’s post hoc test for multiple comparisons: empty vector vs. *Dm*DNMT2: *p* = 0.0016, empty vector vs. *Aa*DNMT2: *p* = 0.0017, *Dm*DNMT2 vs. *Aa*DNMT2: *p* = 0.9971 (Figure 5A).

We next investigated the effect of *Dm*DNMT2 and *Aa*DNMT2 expression on SINV in *Aedes albopictus* C710 cells. Prior studies suggest that viruses and the endosymbiont *Wolbachia* each differentially alter *Aa*DNMT2 expression in the native mosquito host to enhance and restrict virus replication, respectively [7,49]. We, therefore, reasoned that ectopic expression of *Aa*DNMT2 should rescue the virus from *Wolbachia-*mediated inhibition in *Aedes* mosquito cells. At the same time, we also assessed this effect following heterologous expression of *Dm*DNMT2. *Aedes albopictus* (C710)-derived cells (colonized with and without *w*Stri *Wolbachia* strain) were transfected with FLAG-tagged versions of *Dm*DNMT2 or *Aa*DNMT2 and were challenged with SINV at an MOI of 10 particles/cell approximately 72 h post-transfection. As before, cell supernatants were collected after 48 h post-infection, and viral titers were assayed on vertebrate baby hamster kidney fibroblast (BHK-21) cells using standard plaque assays. In line with our hypotheses, expression of *Aa*DNMT2 in cells both colonized with and without *Wolbachia* was associated with significant SINV titer increases (Figure 5B,C). However, we did not find any significant changes in virus titer from cells expressing the non-native *Dm*DNMT2 ortholog; one-way ANOVA with Tukey’s post hoc test for multiple comparisons: Cells without *Wolbachia*, empty vector vs. *Dm*DNMT2: *p* = 0.4788, empty vector vs. *Aa*DNMT2: *p* < 0.01, *Dm*DNMT2 vs. *Aa*DNMT2: *p* < 0.05, Cells *with Wolbachia*, empty vector vs. *Dm*DNMT2: *p* = 0.8705, empty vector vs. *Aa*DNMT2: *p* < 0.05, *Dm*DNMT2 vs. *Aa*DNMT2: *p* < 0.05 (Figure 5B,C). Additionally, we assessed the effect of heterologous *Dm*DNMT2 on viral RNA levels in the cell-based on previous reports that demonstrated the ability of *Aa*DNMT2 to rescue virus replication in the presence of *Wolbachia* [7]. Expression of *Aa*DNMT2 significantly increased SINV RNA levels in *w*Stri-colonized cells. However, heterologous expression of *Dm*DNMT2 did not affect SINV RNA levels, suggesting lack of virus rescue under pathogen blocking conditions; one-way ANOVA with Tukey’s post hoc test for multiple comparisons: SINV RNA, empty vector vs. *Dm*DNMT2: *p* = 0.7875, empty vector vs. *Aa*DNMT2: *p* < 0.05, *Dm*DNMT2 vs. *Aa*DNMT2: *p* < 0.05 (Figure S4A). Finally, to determine whether our observation of virus rescue in the presence of *Wolbachia* was due to changes in endosymbiont titer, we quantified *Wolbachia* titer across our experimental conditions; empty vector vs. *Dm*DNMT2: *p* = 0.4121, empty vector vs. *Aa*DNMT2: *p* = 0.5639, *Dm*DNMT2 vs. *Aa*DNMT2: *p* = 0.9523 (Figure S4B). Altogether, these results suggest that heterologous expression of *Dm*DNMT2 in mosquito cells does not affect virus fitness, although we cannot rule out a dominant effect of the native *Aa*DNMT2 ortholog in mosquito cells or that lower expression of the *Dm*DNMT2 heterologous construct influenced the result. Given the role of *Drosophila*-specific host factor IPOD in regulating *Dm*DNMT2 antiviral function (Figure 2), we speculate that this lack of *Dm*DNMT2 activity in non-native cells (Figure 3E,F) might also be due to the lack of one or more such interaction partners or co-factors of DmDNMT2 in mosquito cells.

## 4. Discussion

This study presents evidence of distinct differences in the structure and regulation of fruit fly and mosquito MTase DNMT2 orthologs that underlie their distinct roles in interaction with viruses in their respective arthropod hosts. This is accompanied by evidence of adaptive evolution of DNMT2 in Dipteran insects that adds support to recent reports describing its role in host innate immunity [6,7,17,18,22,23]. The biological function of DNMT2 remains unexplored in a vast majority of arthropods. Where it has been studied, for example, in *Drosophila melanogaster*, loss of function of DNMT2 is not associated with any severe developmental issues or lethality [39]. Additionally, DNMT2-only insects such as fruit flies and other holometabolous insects exhibit very low to no CpG methylation across their genome, in line with DNMT2′s lack of DNA MTase activity [12]. Recent studies suggest that DNMT2 is part of the cellular stress response that acts against external stressors such as pathogen challenges. Indeed, *Dm*DNMT2 confers protection against a wide range of RNA viruses and bacteria such as *Acetobacter tropocalis*, *Lactobacillus fructivorans*, and *Acetobacter pomorum* [6,17]. Similarly, the DNMT2 ortholog in *Helicoverpa armigera* (Order: Lepidoptera) has been shown to confer protection against systemic infections by *Bacillus thuringiensis* and *Serratia marcescens* [22]. However, there are instances where DNMT2 regulates how well certain pathogens colonize the host in a manner that is seemingly beneficial to the pathogen. Examples of this can be found among members of the Culicidae family [7,23]. In each of these cases, expression of DNMT2 is elevated following an infectious bloodmeal containing either the parasite *Plasmodium berghei (Anopheles albimanus*) or DENV (*Aedes aegypti*) [7,23]. Notably, pharmacological inhibition or miRNA-mediated knockdown of DNMT2 in these species correlates with reduced host susceptibility to infection. Although divergent, it is clear from these examples that Drosophilidae and Culicidae DNMT2 play important roles in shaping their host immune responses to a wide range of pathogens, notably RNA viruses [6,7,17,18,22,23].

### 4.1. Delineating Differences between DNMT2 Regulation in Fruit Fly and Mosquitoes

In addition to the presence or absence of positive selection, we identified two distinct differences in the overall protein sequence between Drosophilidae and Culicidae DNMT2. The first being an extended (7–47 aa in length), unstructured N-terminal end present in all DNMT2 orthologs within Culicidae species. The other difference lies in the TRD, extended (7–11 aa in length) in Drosophilidae DNMT2 and is predicted to interact with the nucleic acid substrate [41]. These differences also give rise to altered surface charge distribution between *Dm*DNMT2 and *Aa*DNMT2, further signifying potential differences in inter-molecular associations and/or target specificity between these orthologs. It could be that unique modes of regulation between the two orthologs are reflected in these differences, a case that is strengthened by our results regarding the role of the *Drosophila melanogaster* protein IPOD in *Dm*DNMT2 regulation. IPOD is present within all members of the Drosophila genus but absent in Culicidae species (Figure S2). Notably, previous in vivo and in vitro analyses indicate that IPOD binds to the N-terminal end of DmDNMT2 [40]. Previous work has also suggested IPOD-mediated regulation of *Dm*DNMT2 expression. Through in vivo loss-of-function analyses, we show that IPOD is indeed an upstream regulator of *Dm*DNMT2 expression. Given that the entirety of IPOD comprises an N-terminal signal peptidase and a C-terminal non-cytoplasmic domain, it is likely that it regulates *Dm*DNMT2 transcription in the nucleus. Finally, demonstrating its functional role in *Dm*DNMT2 regulation, we show that loss of IPOD in flies phenocopies *Dm*DNMT2 loss-of-function mutants [6]. The role of IPOD as a cognate DNMT2 regulator and interaction partner is further supported by our observation that the phylogenies of Drosophila DNMT2 and IPOD orthologs mirror one another to a significant degree, suggesting a co-evolving relationship between these two proteins.

The mechanism of Culicidae DNMT2 regulation is less well defined but likely varies between different mosquito genera. A recent study by Claudio-Piedras et al. suggests that DNMT2 in *Anopheles albimanus* is under the control of the NF-κB family of transcription factors [23]. This contrasts with Aedes mosquitoes, where expression of DNMT2 is under the control of a conserved miRNA *aae-miR-2940* (miRBase Accession: MI0013489) [7]. However, like the miRNA itself, its target mRNA sequence is unique to Aedes DNMT2 and is absent from ortholog transcripts from other Culicidae species and, most notably, from Drosophila DNMT2 (Figure S7A). Still, the absence of this particular miRNA target does not imply that *Dm*DNMT2 is not under the control of any miRNAs. In silico miRNA prediction with *Dm*DNMT2 (FlyBase ID: FBtr0110911) as a target query using miRanda predicts one highly conserved host miRNA, dme-miR-283 (miRBase Accession ID: MI0000368), with the potential of targeting the 3′ untranslated region (3′UTR) of the *Dm*DNMT2 gene. Incidentally, dme-miR-283 is among the top ten most upregulated miRNAs in fly cells following alphavirus (Semilki Forest Virus, SFV) infection, both in the presence and absence of *Wolbachia* [50]. Assuming that dme-miR-283 downregulates *Dm*DNMT2 expression, the modENCODE RNA-seq treatment dataset and our previous observations indicate these results are in line with the SINV-responsive expression pattern of this miRNA and its target in adult flies [6]. It should also be noted that while we found a single miRNA targeting *Dm*DNMT2, miRanda and TargetScanFly v7.2 identified a set of three conserved Drosophila miRNAs targeting the 3′UTR region of multiple Drosophila IPOD orthologs (FlyBase ID: FBgn0030187). A subset of these miRNAs has been previously associated with regulating host innate immunity and antimicrobial responses (Figure S7B) [51]. Further work is necessary to experimentally validate the role of these miRNAs in regulating the expression of their predicted targets.

### 4.2. Influence of Host Backgrounds on DNMT2 Antiviral Activity

**Figure 6:**
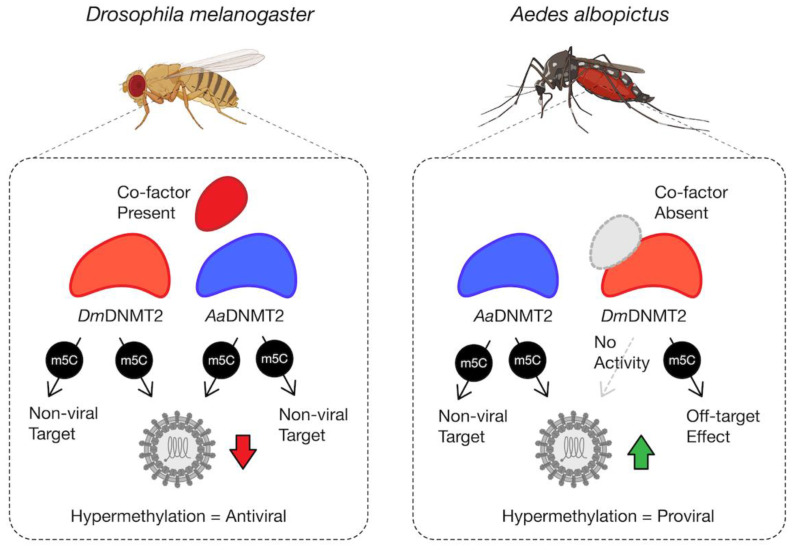
Model schematic of *Dm*DNMT2 and *Aa*DNMT2 activity. Heterologous expression of either *Dm*DNMT2 or *Aa*DNMT2 in *Drosophila melanogaster-*derived JW18 cells leads to virus inhibition, likely as a consequence of hypermethylation of a viral and/or host target. In this case, *Dm*DNMT2 function is potentially aided by the presence of an unidentified co-factor. Heterologous expression of *Aa*DNMT2 in *Wolbachia-*colonized *Aedes albopictus* cells leads to the rescue of virus inhibition, likely due to hypermethylation of a viral and/or host target. In contrast, *Dm*DNMT2 expression in these cells has no observable effect on virus replication, suggesting either a loss in MTase activity or potential off-target effects. This result could be due to the absence of *Dm*DNMT2′s cognate interaction partner(s) or co-factor(s) unique to Drosophila and are thus absent in *Aedes albopictus* cells.

Finally, through heterologous expression of *Dm*DNMT2 and *Aa*DNMT2 in their non-native host backgrounds, we show that the antiviral activity is not unique to *Dm*DNMT2 but is instead a consequence of the host *Drosophila melanogaster* background, as its effect on SINV is phenocopied by heterologous *Aa*DNMT2 expression in the same cells, leading to a loss in infectious virus production (Figure 5A). This suggests that sequence or structural features unique to *Dm*DNMT2 are not responsible for its antiviral activity in fly cells. However, these features indicate the requirement for specific inter-molecular interactions required for proper *Dm*DNMT2 function and specificity. This is supported by our observation that expression of *Dm*DNMT2 in *Aedes albopictus* mosquito cells colonized with or without *Wolbachia* does not affect SINV, either antiviral or proviral, in contrast to the native *Aa*DNMT2 expression, which leads to virus “rescue” from *Wolbachia-*mediated inhibition and improved infectious virus output in the absence of the endosymbiont (Figure 5B,C). We postulate that this complete lack of *Dm*DNMT2 activity and/or specificity in this host (*Aedes albopictus*) background could be due to the absence of one or more *Dm*DNMT2 “co-factors” that are specific to Drosophila, i.e., IPOD (Figure 6).

Our observations regarding *Aa*DNMT2′s ability to function as an antiviral in fly cells suggest that any selection within Drosophila that differs from Aedes may also be due to other adaptations. Still, the sites identified to be under positive selection may contribute to *Dm*DNMT2′s potency as an antiviral. Further work is required to determine whether *Dm*DNMT2 variants carrying the replaced ancestral codons are less efficient at inhibiting viruses native to Drosophila, as they likely represent the source of this selection.

### 4.3. Elucidating the Molecular Evolution of DNMT2

Signatures of positive selection are often a hallmark of genes involved in host immunity. To determine whether DNMT2 itself has been subjected to such selection, we carried out CodeML analyses of DNMT2 orthologs from Dipteran insects, with an increased focus on members of the Culicidae and Drosophilidae families based on their roles in host immunity [28]. We found several instances of positive selection along ancestral and more recent lineages leading to these species, identifying several potential codon sites within each ortholog having experienced positive selection (Figure 4, Table 1 and Table 2). Physiochemical properties and location of these amino acid residues on the 3D structure of *Dm*DNMT2 and *Aa*DNMT2 indicate that these adaptive changes occur in four major protein regions (Figure 4A). Collectively, these changes might influence catalytic function and intermolecular interactions with other accessory proteins and/or nucleic acid substrates. Further work, using site-directed mutagenesis of these sites, is required to validate the importance of these residues on the ability of these DNMT2 orthologs to regulate virus infection. Notably, our CodeML analyses did not find evidence of positive selection along lineages leading to *Aedes* DNMT2 since their divergence with *Anopheles* (Figure 3A). This is in contrast with the antiviral role of *Dm*DNMT2, which could explain the presence of positive selection along this lineage. However, several sites identified in the ancestral Culicidae lineage and related *Anopheles* genera were found to occur within *Aa*DNMT2 (Figure 3A). Furthermore, heterologous expression of this ortholog in fly cells was able to restrict infectious virus production as well as the native *Dm*DNMT2 ortholog, indicating that the outcome is host-dependent (Figure 6). Collectively, our results suggest that several Dipteran DNMT2 orthologs may have evolved to function in host-pathogen interactions, contributing to their antiviral role in fruit flies and possibly other members of the Drosophila genus. Indeed, based on the overall positive selection and complete conservation of these codon sites among Drosophila/Sophophora, it is conceivable that these DNMT2 orthologs confer similar antiviral effects in their respective host backgrounds (Figure 3, Table 1 and Table 2). Given the lack of genetic tractability in these Drosophila species, heterologous expression of these DNMT2 orthologs in a tractable *Drosophila melanogaster* background can be used to determine their restriction properties.

While CodeML has been shown in several studies to be conservative under various conditions, the authors of hyphy have shown CodeML to produce false positives under somewhat extreme conditions where rate variation among the background branches leads to a strong violation of model assumptions [52,53,54,55,56]. In particular, their RS1 simulation, which sets the foreground branch to ω = 1, two background branches to ω = 10 and two other background branches to ω = 0.1, caused CodeML to give significant predictions of ω > 1 for the neutral foreground branch, which worsened with increasing sequence length. There is undoubtedly dN/dS rate variation in our DNMT2 data, but whether it is extreme enough to lead to many false positives is uncertain. The hyphy method, aBSREL, which tries to account for such variance, does find fewer branches with raw *p*-value < 0.05 (Table 1, Nodes 20, 30, 35, 54, 66). It is probable that some of the CodeML *p*-values < 0.05 are due to some model violation, and many of these raw *p*-values are not significant after correcting for multiple tests but finding so many branches that point to positive selection is unusual and does give the impression that positive selection, which is hard to detect, is prevalent in this gene.

Since the exact mechanism of DNMT2′s antiviral role remains undefined, it is possible that these adaptations allow for functional differences of this MTase against specific viruses, host conditions, or both. Recent evidence suggests the presence of *Wolbachia* in *Aedes albopictus* cells is associated with hypomethylation of SINV virion encapsidated RNA, which is correlated with reduced *Aa*DNMT2 expression implicating this MTase as a mediator of pathogen blocking. Still, our data suggest fundamental differences between mosquito and fly cells regarding the effect of native DNMT2 on viral infection. Given that the RNA virus used in this study belong to the alphavirus family, which are native to the *Aedes* host, the antiviral activity of both MTase orthologs against SINV in fly cells could therefore also be due to fundamental differences in the host response to potential hypermethylation of viral and host RNA species. Indeed, while such modifications may be favorable or even necessary for alphavirus replication in the native mosquito, they might allow for virus recognition and clearance in the fly background. Further studies are required using native virus-host-MTase ortholog combinations to explore these possibilities. At the same time, based on our current experimental setup, we cannot rule out the possibility that basal-level expression of the endogenous MTase affects the outcomes of our heterologous-expression experiments. Further work is required to determine whether heterologous expression of *Aa*DNMT2 can complement the absence of the native-*Dm*DNMT2 null fly cells, and vice versa, with regard to virus restriction or rescue respectively.

## 5. Conclusions

Collectively, in this study, we report a broad role of the DNA/RNA cytosine MTase DNMT2 as an immune factor in Dipteran insects. More specifically, we provide evidence of the rapid evolution of this protein, identifying specific amino acid residues in Culicidae and Drosophila DNMT2 orthologs that might signify unique inter-molecular interactions that might influence their distinct roles in virus regulation. These interactions include at least one regulatory Drosophila protein, IPOD, which we show is an upstream regulator of *Dm*DNMT2 expression and activity. Using the heterologous expression of *Dm*DNMT2 in mosquito cells, we demonstrate a potential requirement of this and other yet unidentified “co-factors” for proper MTase function. In contrast, our results indicate that *Aa*DNMT2 is functional in fly cells. However, its effect on virus production, either pro- or antiviral, is dependent on the Drosophila host background, thus indicating fundamental differences between the two arthropod models in terms of the functional consequence of potential cytosine methylation on the outcome of RNA virus infection.

## Figures and Tables

**Table 1:** CodeML analyses result of positive selection among DNMT2 orthologs. The column “Amino Acid Sites” shows codon positions with BEB posterior probability > 0.80 for having ω > 1. Underlined codon sites represent those with BEB posterior probability > 0.95.

Species	2lnλ	*p-*Value	Amino Acid Sites	Branch (No. of Taxa)
***All Dipteran species***	5.4	0.01	44G, 55S	3 (20)
***Culex quinquefasciatus***	18.7	0.004370	17E, 24K, 46N, 323S	54 (1)
***Anopheles dirus***	2.9	0.044	274L	28 (1)
***Anopheles darlingi*** ***Anopheles albimanus***	8.7	0.002	248S, 263E	21 (2)
***Anopheles* sub-genus**	11.0	0.001445	13H, 14F	20 (15)
***Culicidae* Species Family**	10.1	0.0007	84F, 103D, 105I, 147H, 208K, 222C,309C, 328E	19 (17)
***Anopheles minimus*** ***Anopheles culicifacies*** ***Anopheles funestus*** ***Anopheles stephensi***	9.4	0.036654	24K	30 (4)
***Anopheles minimus***	4.0	0.02	-	35 (1)
***Anopheles gambiae*** ***Anopheles coluzzi***	5.0	0.012	-	42 (2)
***Anopheles gambiae* sub-genus**	3.5	0.009	-	25 (12)
***Drosophila melanogaster*** ***Stomoxys calcitrans*** ***Musca domestica*** ***Glossina* sp. **	6.1	0.007	23Y, 78F	4 (8)
***Drosophila melanogaster***	4.2	0.02	223T, 226S, 228S, 255F	5 (1)
***Glossina* sp. **	7.6	0.003	100D, 150G, 214K	10 (5)
***Stomoxys calcitrans*** ***Musca domestica*** ***Glossina* sp. **	8.8	0.001	51S, 55S, 123Q, 208K	6 (7)
***Stomoxys calcitrans*** ***Musca domestica***	3.2	0.04	26V	7 (2)

**Table 2:** CodeML analyses result of positive selection among Drosophilid DNMT2 orthologs. The column “Amino Acid Sites” shows codon positions with BEB posterior probability > 0.95 for having ω > 1. *Drosophila melanogaster* taxa and associated amino acid sites are represented in bold. The codon sites within parenthesis relate to the positions for the same sites on the Dipteran multiple sequence alignment used in CodeML analyses for Table 1 and Figure 3A.

Species	2lnλ	*p-*Value	Amino Acid Sites	Branch (No. of Taxa)
*Drosophila biarmipes* *Drosophila suzukii* *Drosophila takahasii* *Drosophila rhopaloa* *Drosophila elegans* *Drosophila bipectinata* *Drosophila ananassae* *Drosophila ficusphila* *Drosophila eugracilis* *Drosophila simulans* *Drosophila melanogaster* *Drosophila erecta* *Drosophila teisseri* *Drosophila yakuba* *Drosophila kikkawai* *Drosophila pseudoobscura* *Drosophila obscura* *Drosophila willistoni*	8.2	0.002	90**(87)**T, 263**(261)**L, 325**(320)**K	41 (18)
*Drosophila mojavensis* *Drosophila arizonae* *Drosophila navajoa* *Drosophila hydei* *Drosophila gaucha* *Drosophila gasici* *Drosophila imcompta* *Drosophila novamexicana* *Drosophila virilis* *Drosophila busckii* *Drosophila grimshawi* *Drosophila maculifrons* *Drosophila griselolineata* *Drosophila mediodiffusa* *Drosophila mediostriata* *Drosophila nappae* *Drosophila omatifrons* *Drosophila subbadia* *Drosophila guaru* *Drosophila tripunctata*	3.7	0.02	96D	2 (20)
*Drosophila bipectinata*	5.2	0.01	182M, 196A	64 (1)
*Drosophila ficusphila*	3.0	0.04	179W	66 (1)
*Drosophila teisseri*	6.1	0.007	58S	70 (1)
*Drosophila grimshawi*	3.3	0.035	107E	23 (1)

## Data Availability

All the experimental data presented in this manuscript can be found in the publication and the supplementary information.

## Supplementary Materials

The following are available online at https://www.mdpi.com/article/10.3390/v13081464/s1, Figure S1: Amino-acid positions in Culicidae and Drosophila DNMT2 under positive selection. Figure S2: Amino acid sites under positive selection in Anopheles; Figure S3: Differences in primary amino acid sequence between Culicidae and Drosophila DNMT2 orthologs. Figure S4: Presence of IPOD orthologs across Dipterans. Figure S5: Relative Wolbachia titer in IPOD knockdown flies. Figure S6: Effect of heterologous DNMT2 expression on Wolbachia in mosquito cells. Figure S7: Predicted role of miRNAs in regulation of Drosophila DNMT2 and IPOD. Table S1: Primers used in this study.
