# Supplementary material for: Evidence of Adaptive Evolution in Wolbachia-Regulated Gene DNMT2 and Its Role in the Dipteran Immune Response and Pathogen Blocking
Source: Viruses. 2021 Jul 27;13(8):1464. doi: 10.3390/v13081464 (PMC8402854; doi:10.3390/v13081464)
Supplement: Supplementary file 1 [file viruses-13-01464-s001.zip › Supplemental Figures/Supplementary Table 2.pdf]

IPOD Ortholog Sequence Organism	Accession ID
Musca domestica	XM_005185833.3
Lucilia cuprina	XM_023441743.1
Sarcophaga Bullata	TMW51081.1
Drosophila busckii	XM_017994561.1
Drosophila albomicans	XM_034246300.1
Drosophila innubila	XM_034626961.1
Drosophila grimshawi	XM_001991945.2
Drosophila novamexicana	XM_030708971.1
Drosophila virilis	XM_032439763.1
Drosophila hydei	XM_030225904.1
Drosophila navojoa	XM_030390557.1
Drosophila mojavensis	XM_032733185.1
Drosophila arizonae	XM_018013864.1
Drosophila guanche	XM_034267725.1
Drosophila obscura	XM_022378499.1
Drosophila miranda	XM_033396128.1
Drosophila pseudoobscura	XM_002136795.3
Drosophila persimilis	XM_002022551.2
Drosophila willistoni	XM_002071709.3
Drosophila bipectinata	XM_017239095.1
Drosophila ananassae	XM_032452351.1
Drosophila takahashii	XM_017157359.1
Drosophila biarmipes	XM_017098955.1
Drosophila suzukii	XM_017081327.1
Drosophila ficusphila	XM_017199699.1
Drosophila rhopaloa	XM_017131156.1
Drosophila erecta	XM_026982714.1
Drosophila yakuba	XM_015189180.1
Drosophila eugracilis	XM_017218214.1
Drosophila simulans	XM_016180916.1
Drosophila mauritiana	XM_033314490.1

Drosophila sechellia	XM_032725577.1
Drosophila melanogaster	NM_132386.3
Drosophila elegans	XM_017259381.1
Drosophila serrata	XM_020946921.1
Drosophila kikkawai	XM_017174623.1
