# Supplementary material for: Evidence of Adaptive Evolution in Wolbachia-Regulated Gene DNMT2 and Its Role in the Dipteran Immune Response and Pathogen Blocking
Source: Viruses. 2021 Jul 27;13(8):1464. doi: 10.3390/v13081464 (PMC8402854; doi:10.3390/v13081464)
Supplement: Supplementary file 1 [file viruses-13-01464-s001.zip › Supplemental Figures/viruses-1022112-supplementary.pdf]

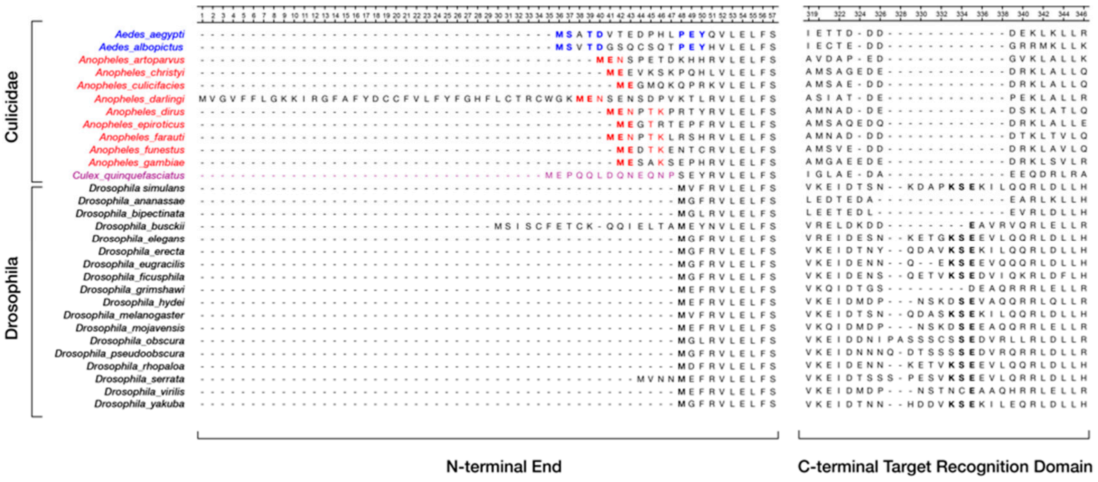

Supplementary Figure S1: Differences in primary amino acid sequence between Culicidae and Drosophila DNMT2 orthologs. Conserved amino acids present in the extended N-terminal end and the C-terminal Target Recognition Domains of Culicidae Drosophila species represented in a multiple sequence alignment, with Aedes genera in blue, Anopheles genera in red and Culex in purple. Fully conserved residues are in represented in bold letters.

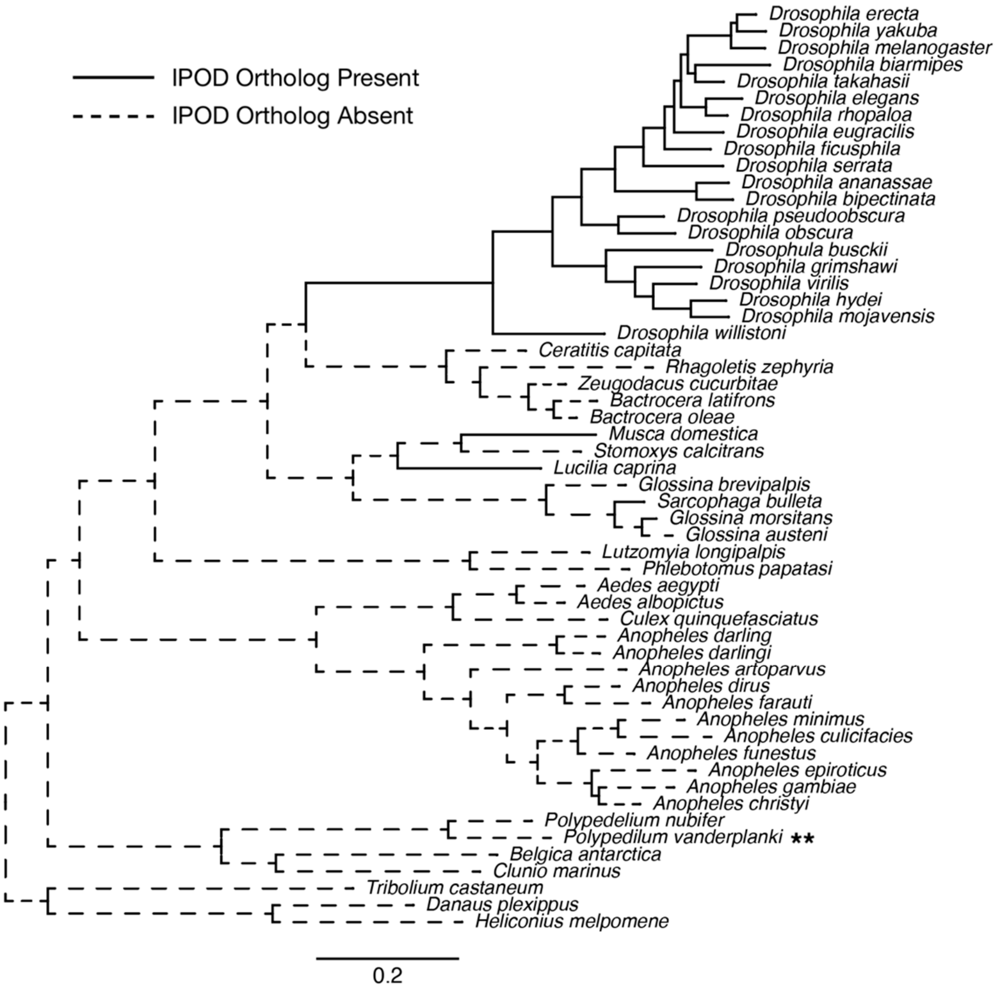

Supplementary Figure S2: Presence of IPOD orthologs across *Dipterans*. *Dipteran* species with known IPOD orthologs (Protein-BLAST) represented on Maximum-likelihood (42) tree constructed

using DNMT2 sequences in RAxML. As presented in Figure 4B, IPOD orthologs are present within *Drosophilidae* and only three other *Dipteran* species (represented in this tree in solid branches). Protein-BLAST failed to identify any potential IPOD orthologs in other *Dipteran* species (represented with dashed branches). Taxa label with accompanying asterisks (**) represent the lack of a full genome assembly and therefore should not be considered while interpreting the results. Scale bar represent branch lengths.

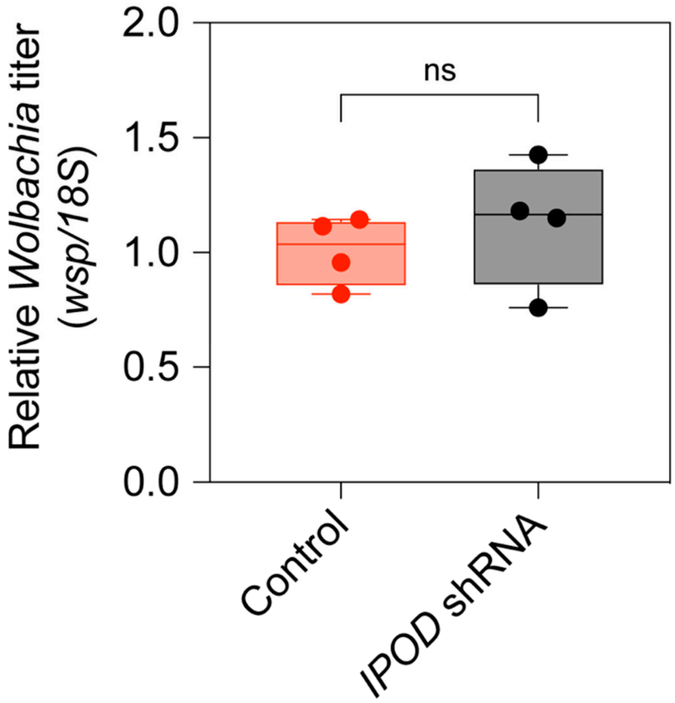

Supplementary Figure S3: Relative *Wolbachia* titer in *IPOD* knockdown flies. Quantitative RT-PCR was used to measure expression of the *Wolbachia wsp* gene relative to the endogenous ribosomal 18S RNA in age-matched 2-4 days old adult female flies following RNAi-mediated knockdown of *IPOD*. *IPOD* expression was knocked down in *Wolbachia w*Mel-colonized *Drosophila melanogaster* (TRiP line# 60092*)* by driving expression of a targeting short-hairpin RNA (shRNA) against the target *IPOD* mRNA. Controls represent isogenic sibling flies not expressing the targeting shRNA. Unpaired Welch's t-test: p = 0.4788, t = 0.7695, df = 4. Error bars represent standard error of mean of 4 independent experimental replicates (consisting of one fly each). ns = not

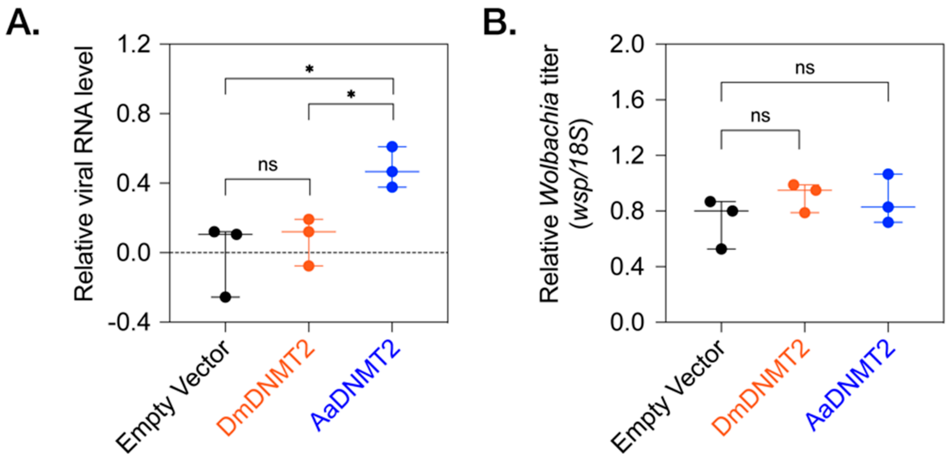

Supplementary Figure S4: Effect of heterologous DNMT2 expression on *Wolbachia* in mosquito cells. 72 hours post transfection, *Aedes albopictus* derived C710 cells (colonized with *w*Stri

Wolbachia strain) expressing either the empty vector, the native DNMT2 (*Dm*DNMT2) or the non-native DNMT2 (*Aa*DNMT2) were challenged with SINV at MOI of 10 particles/cell. Cell lysates were collected 48 hours post infection and levels of (A) virus and (B) *Wolbachia* RNA levels were assessed using qRT-PCR on total extracted RNA. One-way ANOVA with Tukey's post hoc test for multiple comparisons: SINV RNA, Empty Vector vs *Dm*DNMT2: $p = 0.7875$, Empty Vector vs *Aa*DNMT2: $p < 0.05$, *Dm*DNMT2 vs *Aa*DNMT2: $p < 0.05$, *Wolbachia*, Empty Vector vs *Dm*DNMT2: $p = 0.4121$, Empty Vector vs *Aa*DNMT2: $p = 0.5639$, *Dm*DNMT2 vs *Aa*DNMT2: $p = 0.9523$. Error bars represent standard error of mean of 3 independent experiments. *$p < 0.05$, ns = not-significant. The data presented in panels A and B are representative of independent infection experiments performed in triplicate.

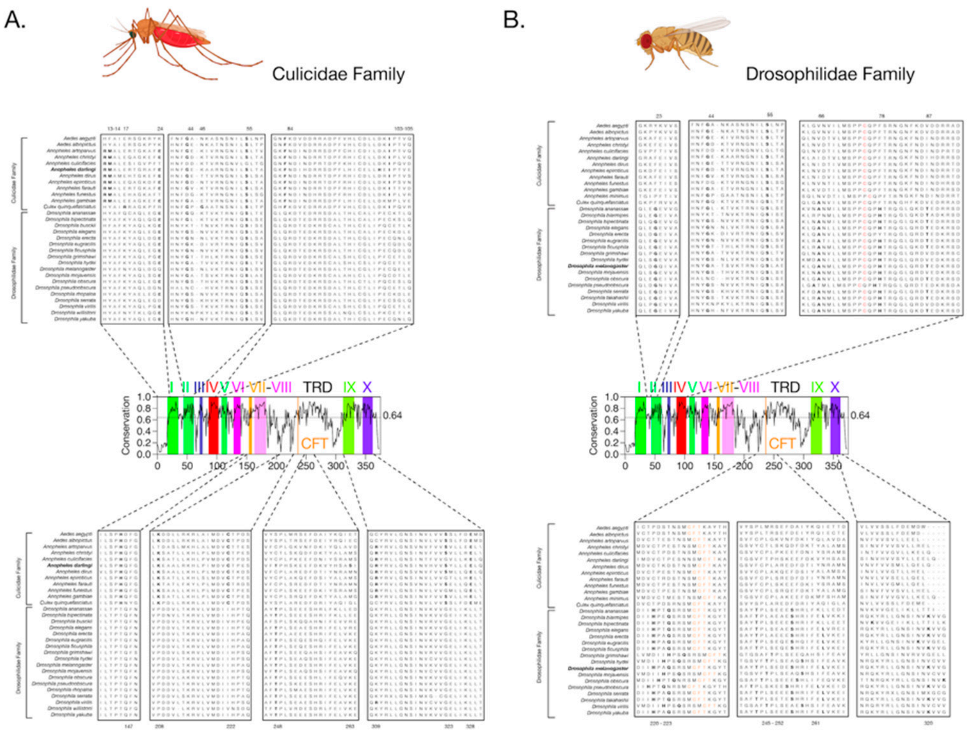

Supplementary Figure S5. Amino-acid positions in Culicidae and Drosophila DNMT2 under positive selection. The global degree of primary sequence conservation (Shannon Conservation Score, Y-axis) of DNMT2 orthologs present at every amino acid position (X-axis) across *Drosophila* and *Culicidae* species. Colored boxes represent a total of ten canonical sequence motifs conserved within eukaryotic DNMT2, in addition to the DNA/RNA binding CFT motif located in the otherwise variable DNMT2 target recognition domain (TRD). Horizontal dotted line and the associated number on the left represent the mean Shannon conservation score averaged across all amino acid positions. Conservation of the amino acid positions in DNMT2 orthologs across *Drosophilids* and *Culicidae* species. Multiple sequence alignment of DNMT2 amino acid sequences was performed using U-Gene. Potential evidence of positive selection was identified along several branches (Figure 1, Table 1). Associated amino acid positions with high posterior probability values (> 95%) were considered as sites under selection (see Tables 1 and 2 for details) and are represented in bold letters. Catalytic Cysteine (Cys, C) residue within Motif IV is represented in red, bold letters. DNA/RNA binding CFT Motif is represented in orange, bold letters.

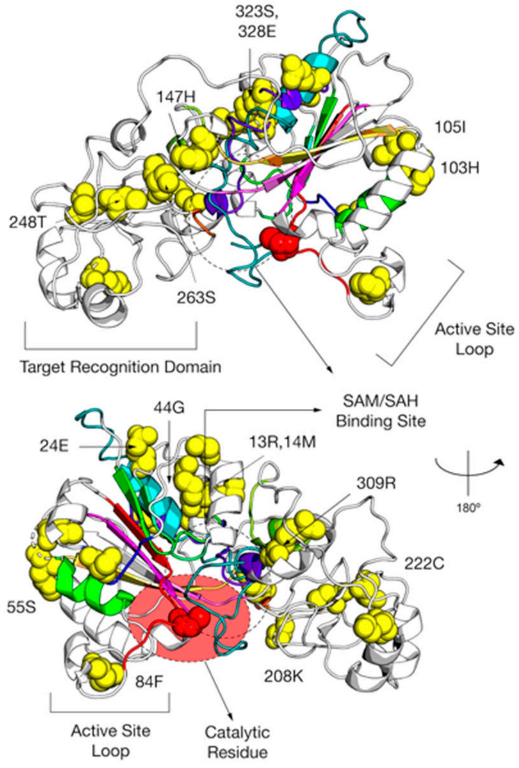

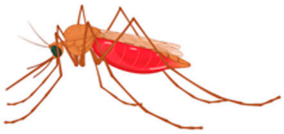

Supplementary Figure S6. Amino acid sites under positive selection in *Anopheles*. Amino acid positions with evidence of positive selection and high posterior probability values (> 95%) were identified within DNMT2 orthologs from mosquitoes (Culicidae, Figure 1A, Table 1). Spatial distribution of 16 sites unique to *Anopheles darlingi*, which include sites present in ancestral branches (3,19,20,21, Table 1) are represented as yellow spheres on ribbon model of *Anopheles darlingi* DNMT2 structure visualized in PyMOL 2.4 (Schrödinger, LLC). Catalytically active cysteine residue (Cys, C) is represented in red. Predicted substrate i.e., S-adenosyl methionine (SAM) or S-adenosyl homocysteine (SAH) binding sites are indicated in oval with dashed outline. Functionally important active-site loop and target recognition domain are also indicated on each structure. The rotation symbol reflects structural features viewed 180º apart along the vertical axis.

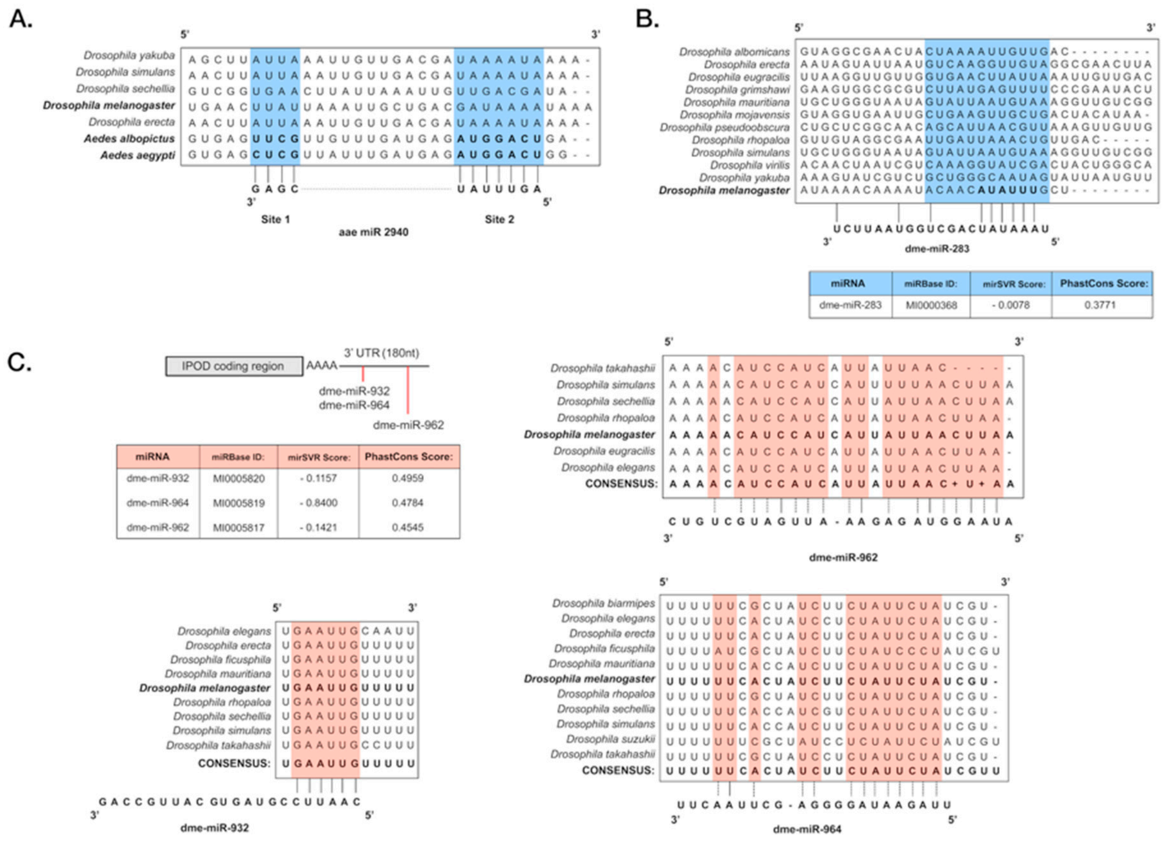

Supplementary Figure S7: Predicted role of miRNAs in regulation of *Drosophila* DNMT2 and IPOD. (A) Prior studies demonstrate the role of the *Aedes* miRNA *aae-miR-2940-5p* in regulating the expression of *Aedes* DNMT2 orthologs (Zhang et.al. 2013). Target sequence for this miRNA is conserved at the primary nucleotide sequence level in *Aedes albopictus* and *Aedes aegypti* DNMT2 *(Mt2)* mRNAs (indicated in the multiple sequence alignment) but absent in *Mt2* mRNAs of orthologs present in all *Drosophila* species, including *Drosophila melanogaster* (taxa depicted in bold letters). (B) Location of conserved miRNA dme-miR-283 predicted to target the 3′ untranslated region (3′UTR) of *Drosophila melanogaster* DNMT2 (taxon depicted in bold letters). (C) Location of conserved miRNAs predicted using mirSVR (microrna.org) to target the 3′ untranslated region (3′UTR) of IPOD. Empirical probability of target downregulation for each miRNA, considering the conservation of the target site, is indicated by the mirSVR downregulation scores. Evolutionary conservation of each of the miRNA target sequences is indicated by the PhastCons scores (PHylogenetic Analysis with Space/Time models CONServation). (C) Sequence conservation of miRNA target region(s) are depicted in light red within aligned nucleotide sequences of 3′UTR regions belonging to IPOD orthologs of different *Drosophila* species. Nucleotide sequence(s) of *Drosophila melanogaster* IPOD is depicted in bold letters.

Primer Name	Forward Primer Sequence (5'-3')	Reverse Primer Sequence (5'-3')
SINV E1	TCAGATGCACCACTGGTCTCAACA	ATTGACCTTCGCGGTCGGATACAT
18S	CGAAAGTTAGAGGTTCGAAGGCGA	CCGTGTTGAGTCAAATTAAGCCGC
WSP	CATTGGTGTTGGTGTTGGTG	ACCGAAATAACGAGCTCCAG
IPOD	CTGCTCCCATTGCCTATCAT	TAACCATGTCCCGAAGCATAC
Dnmt2	CCGTGGCGTGAAATAGCG	ACACCGCTTTCGGAGGACG
pAFW-Mt2 QC_SalI	ACAAGGATGACGATGACAAGGTCCGAC	GGGTCGGCGCGCCCACCCTTGTCGAC
pAFW-Mt2_GA_Insert	AGGATGACGATGACAAGGTCATGGTATTTCGGGTCTTAGA	TCGGCGCGCCCACCCTTGTCTCATTTTATCGTCAGCAATT
pAFW-AMt2	GCAACCGGTTTATGAGTGTTACCGACGGA	GCAGCTAGCTCAGTCCATCTCATCAAACAACGAACTC

Supplementary Table S1. Primers used in this study. Primers were purchased from Integrated DNA Technologies (IDT). All primers were used at a final concentration of 10μM for PCR and quantitative RT-PCR reactions.
