# Supplementary figures and images for: Evidence of Adaptive Evolution in Wolbachia-Regulated Gene DNMT2 and Its Role in the Dipteran Immune Response and Pathogen Blocking
Source: Viruses. 2021 Jul 27;13(8):1464. doi: 10.3390/v13081464 (PMC8402854; doi:10.3390/v13081464)

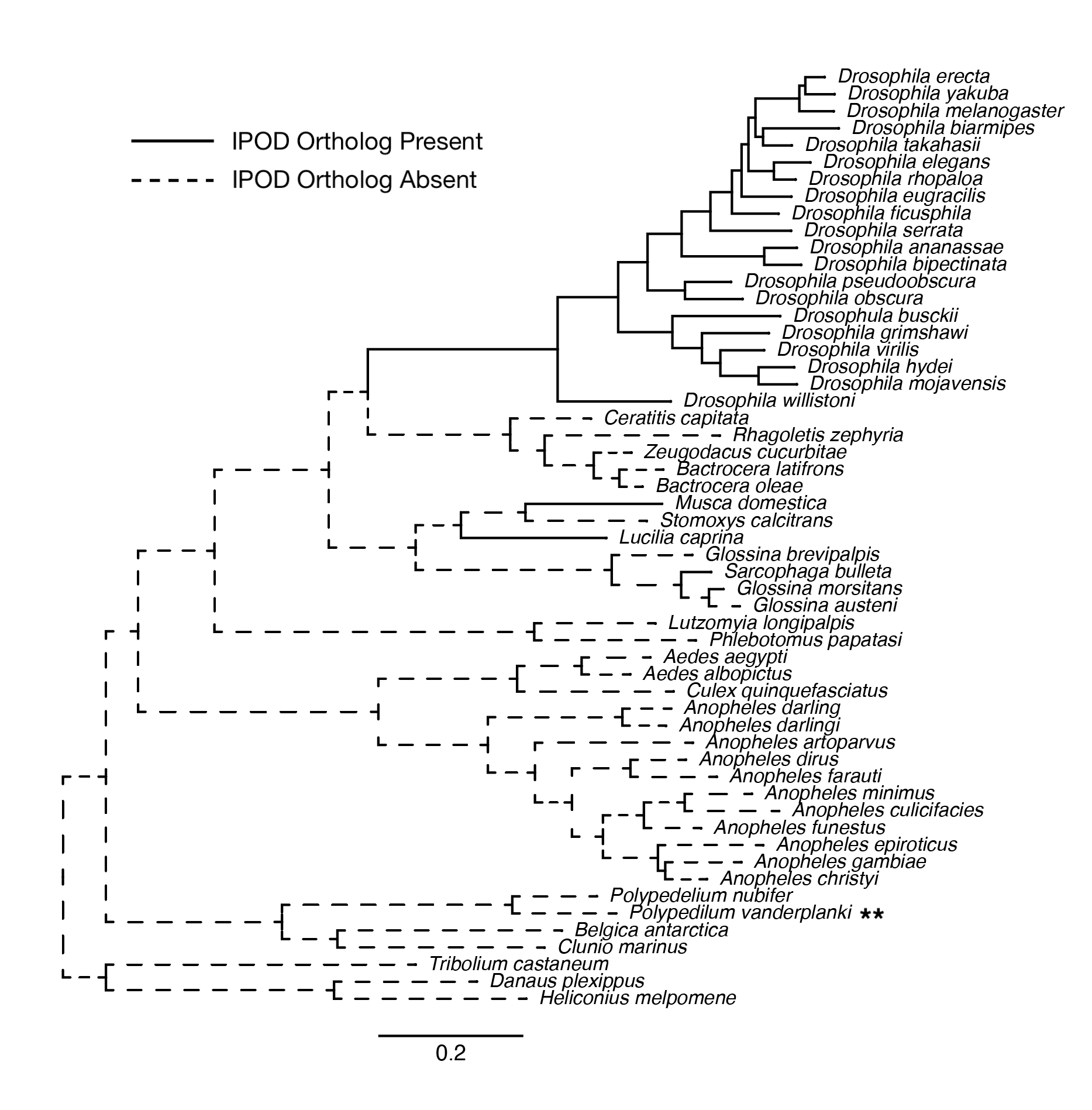

Supplement: Supplementary file 1 [file viruses-13-01464-s001.zip › Supplemental Figures/Supplemental_2_IPOD_Presence Absence.tiff]

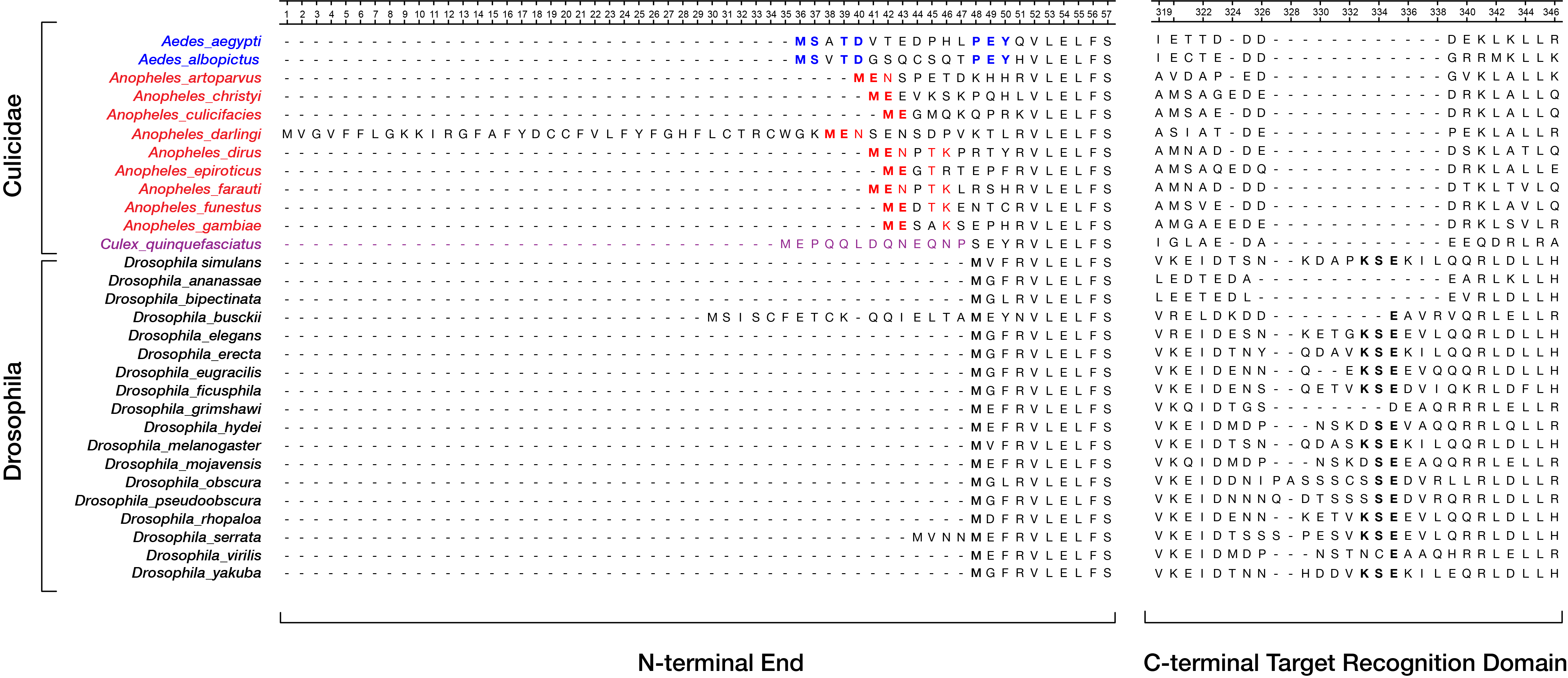

Supplement: Supplementary file 1 [file viruses-13-01464-s001.zip › Supplemental Figures/Supplementary Figure_1.tif]

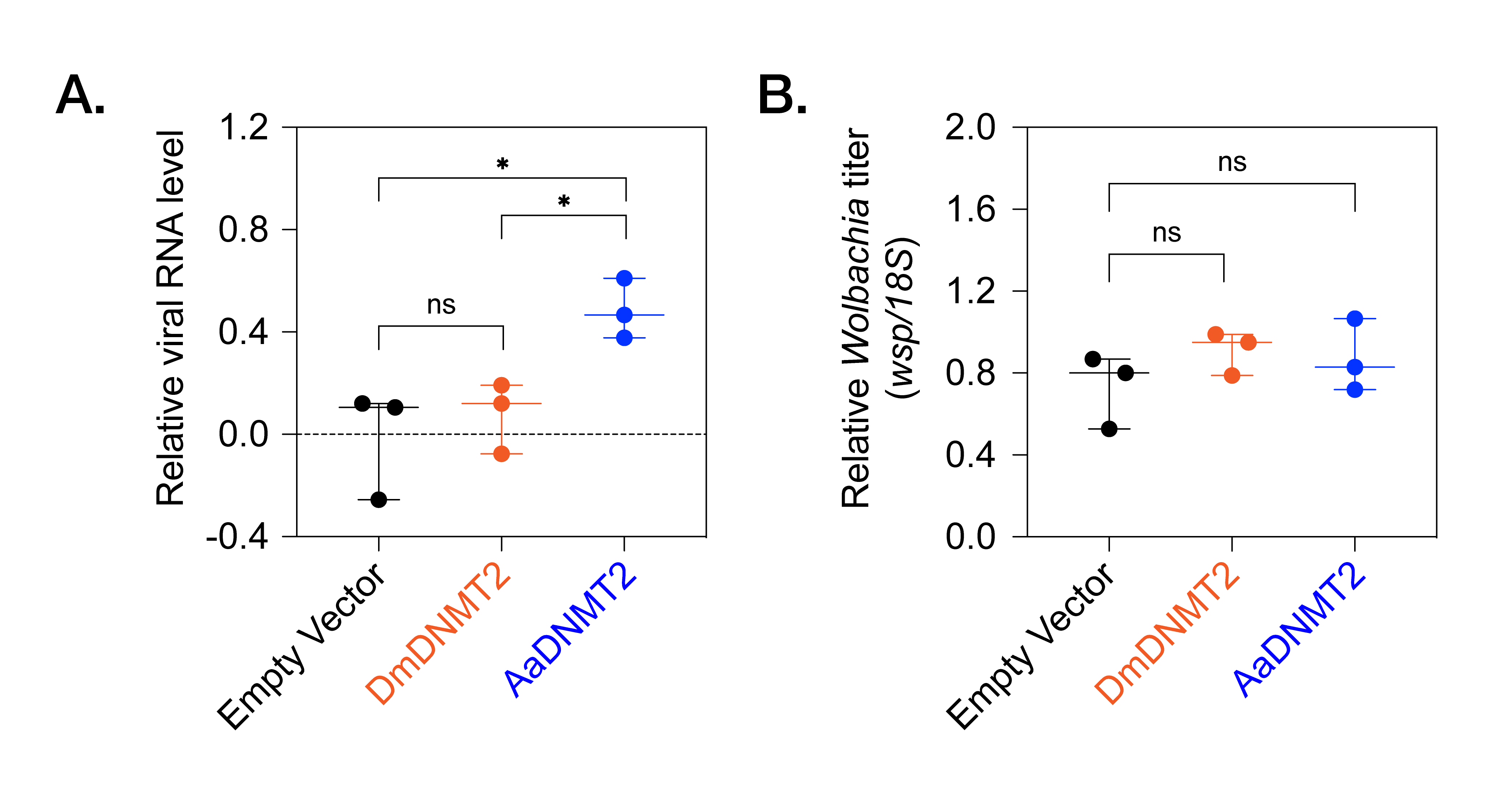

Supplement: Supplementary file 1 [file viruses-13-01464-s001.zip › Supplemental Figures/Supplementary Figure_4_ C710 wStri virus Wolbachia RNA.tif]

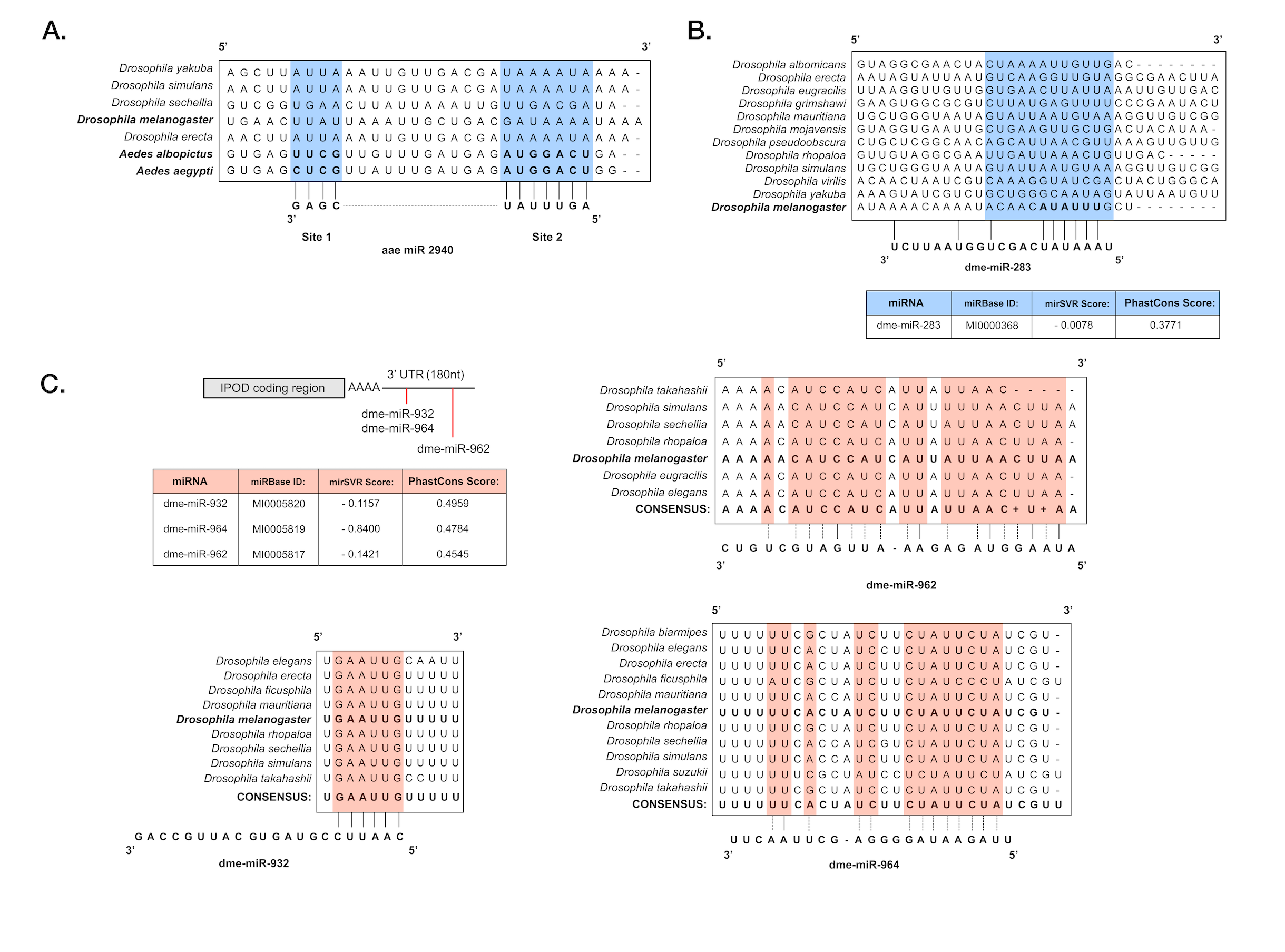

Supplement: Supplementary file 1 [file viruses-13-01464-s001.zip › Supplemental Figures/Supplementary Figure_7_miRNA regulation.tif]

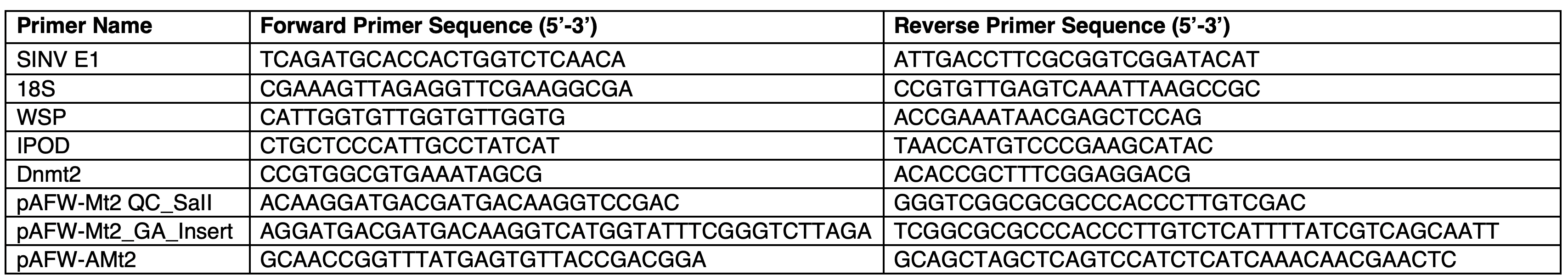

Supplement: Supplementary file 1 [file viruses-13-01464-s001.zip › Supplemental Figures/Supplementary Table 1_Primer Table.tif]

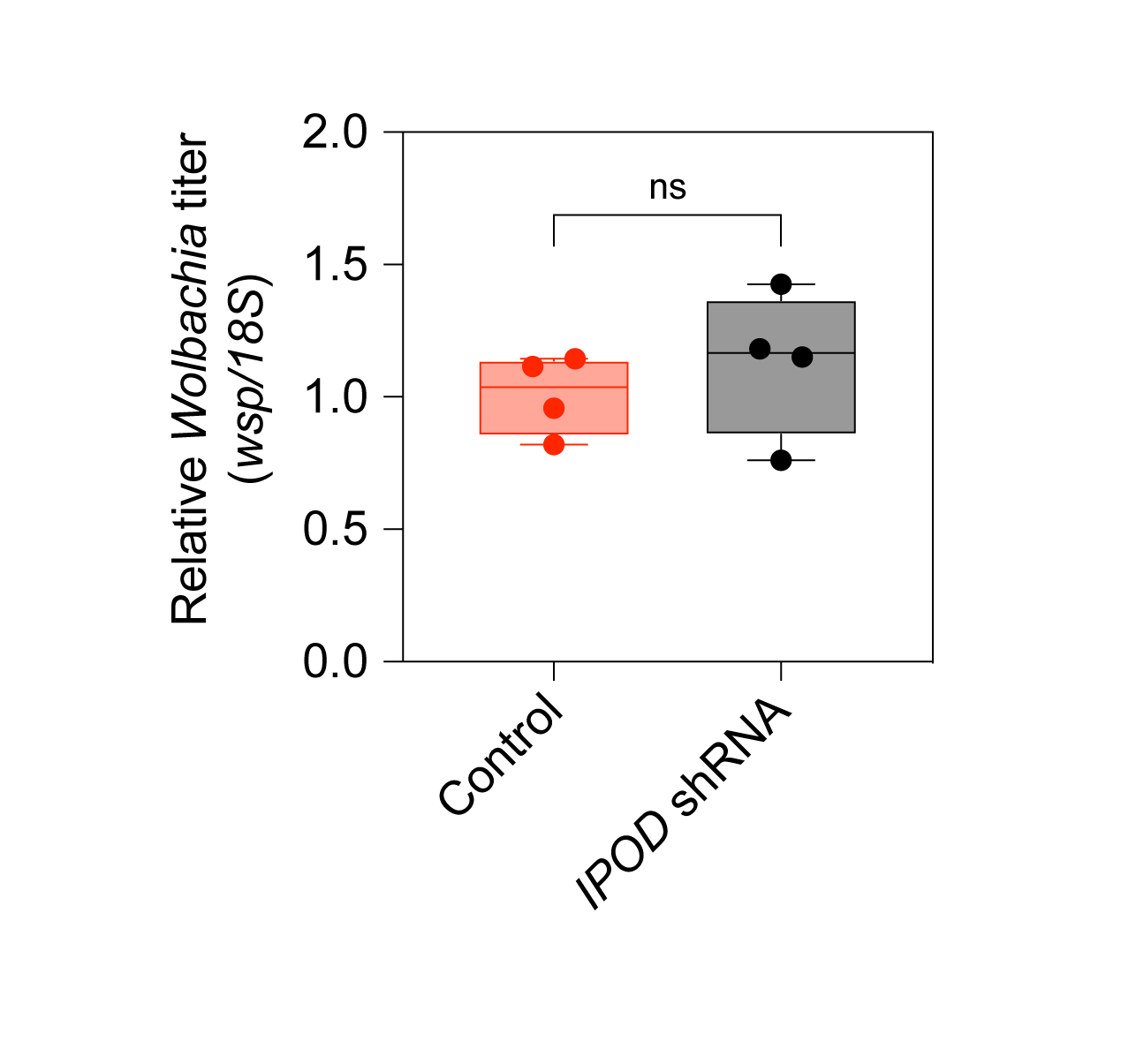

Supplement: Supplementary file 1 [file viruses-13-01464-s001.zip › Supplemental Figures/Supplementary_3_IPOD_Wolbachia titer.tif]

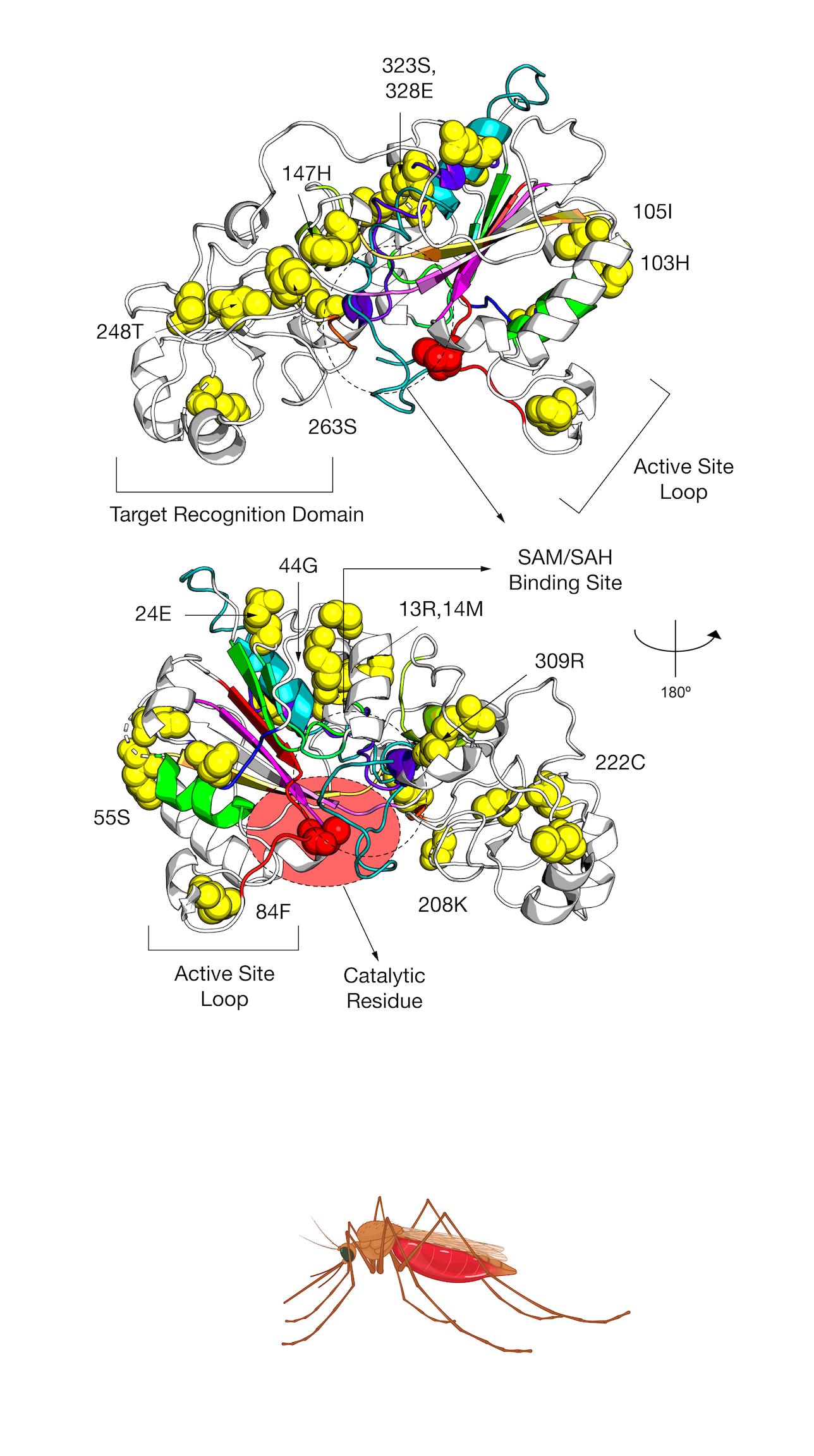

Supplement: Supplementary file 1 [file viruses-13-01464-s001.zip › Supplemental Figures/Supplementary_Figure 6_Anopheles_Structure_Positive Selection Sites_7-1-2020.tif]

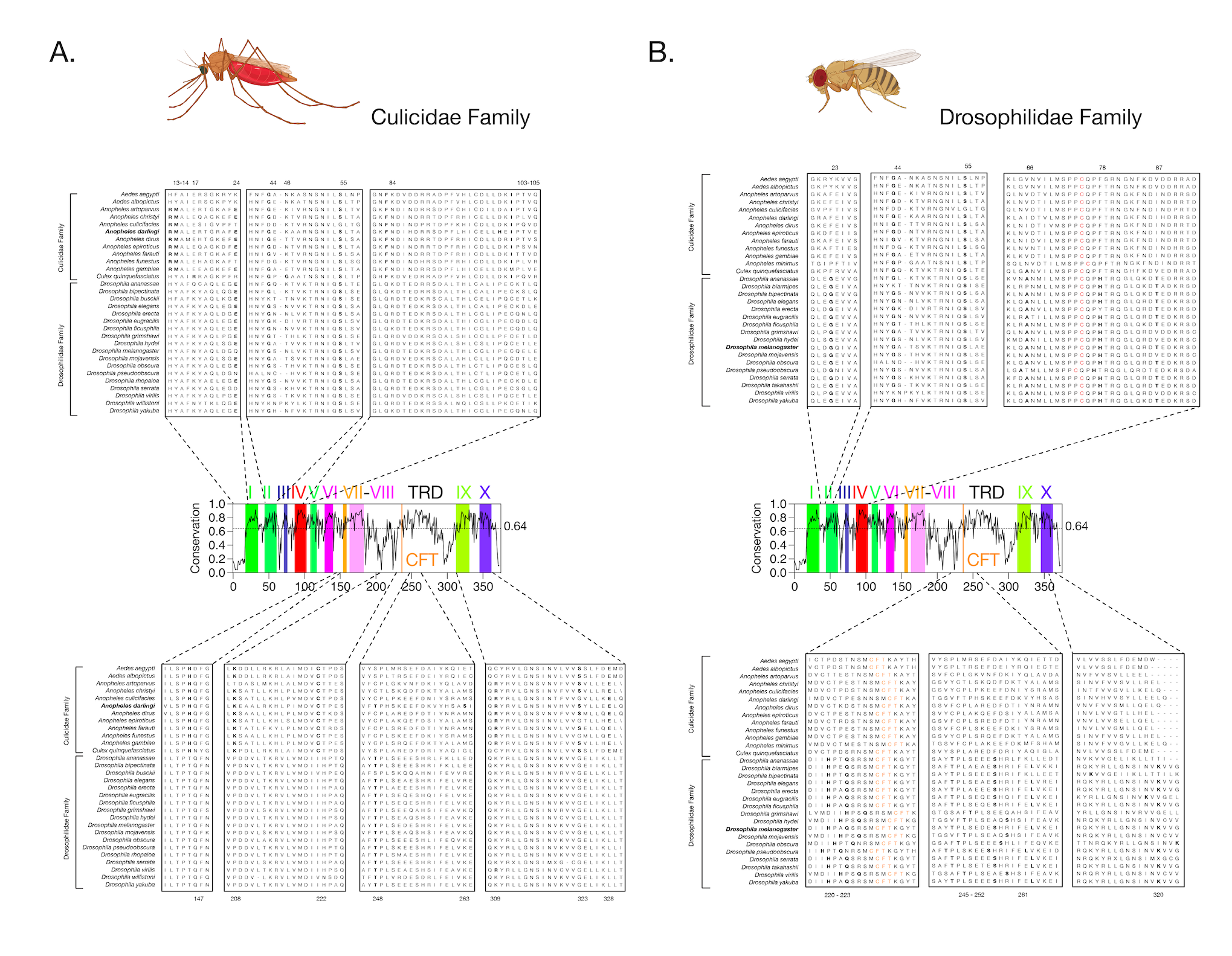

Supplement: Supplementary file 1 [file viruses-13-01464-s001.zip › Supplemental Figures/Supplmentary Figure 5_Drosophila+Culicidae Sites_7-1-2020.tif]
